# Co-factor independent oxidases ncnN and actVA-3 are involved in the dimerization of benzoisochromanequinone antibiotics in naphthocyclinone and actinorhodin biosynthesis

**DOI:** 10.1093/femsle/fnad123

**Published:** 2023-11-21

**Authors:** Bikash Baral, Soheila Matroodi, Vilja Siitonen, Keshav Thapa, Amir Akhgari, Keith Yamada, Aleksi Nuutila, Mikko Metsä-Ketelä

**Affiliations:** Department of Life Technologies, University of Turku, FIN-20014 Turku, Finland; Department of Life Technologies, University of Turku, FIN-20014 Turku, Finland; Laboratory of Biotechnology, Department of Marine Biology, Faculty of Marine Science and Oceanography, University of Marine Science and Technology, 64199-34619 Khorramshahr, Iran; Department of Life Technologies, University of Turku, FIN-20014 Turku, Finland; Department of Life Technologies, University of Turku, FIN-20014 Turku, Finland; Department of Life Technologies, University of Turku, FIN-20014 Turku, Finland; Department of Life Technologies, University of Turku, FIN-20014 Turku, Finland; Department of Life Technologies, University of Turku, FIN-20014 Turku, Finland; Department of Life Technologies, University of Turku, FIN-20014 Turku, Finland

**Keywords:** antibiotics, chemical diversity, natural products, recombinant polyketides, signalling cascade

## Abstract

*Streptomyces* produce complex bioactive secondary metabolites with remarkable chemical diversity. Benzoisochromanequinone polyketides actinorhodin and naphthocyclinone are formed through dimerization of half-molecules via single or double carbon-carbon bonds, respectively. Here we sequenced the genome of *S. arenae* DSM40737 to identify the naphthocyclinone gene cluster and established heterologous production in *S. albus* J1074 by utilizing direct cluster capture techniques. Comparative sequence analysis uncovered *ncnN* and *ncnM* gene products as putative enzymes responsible for dimerization. Inactivation of *ncnN* that is homologous to atypical co-factor independent oxidases resulted in the accumulation of fogacin, which is likely a reduced shunt product of the true substrate for naphthocyclinone dimerization. In agreement, inactivation of the homologous *act*VA-3 in *S. coelicolor* M145 also led to significantly reduced production of actinorhodin. Previous work has identified the NAD(P)H-dependent reductase ActVA-4 as the key enzyme in actinorhodin dimerization, but surprisingly inactivation of the homologous *ncnM* did not abolish naphthocyclinone formation and the mutation may have been complemented by an endogenous gene product. Our data suggests that dimerization of benzoisochromanequinone polyketides require two-component reductase-oxidase systems.

## Teaser

Here, we have elucidated how antibiotic producing bacteria are able to synthesize complex natural products using highly sophisticated chemical transformations.

## Introduction

*Streptomyces* are soil-dwelling prokaryotes with a capability to generate numerous bioactive secondary metabolites that are harnessed for medical usage as antibiotics, anticancer agents and immunosuppressants (Newman and Cragg 2020). A key feature of microbial natural products is their chemical complexity and diversity. Surprisingly, much of this chemodiversity is generated via a limited number of biosynthetic systems that classify secondary metabolites to the main classes of polyketides, non-ribosomal peptides, ribosomally synthesized, and post-translationally modified peptides, and terpenes (Fewer and Metsä-Ketelä 2020).

**Figure 1.**
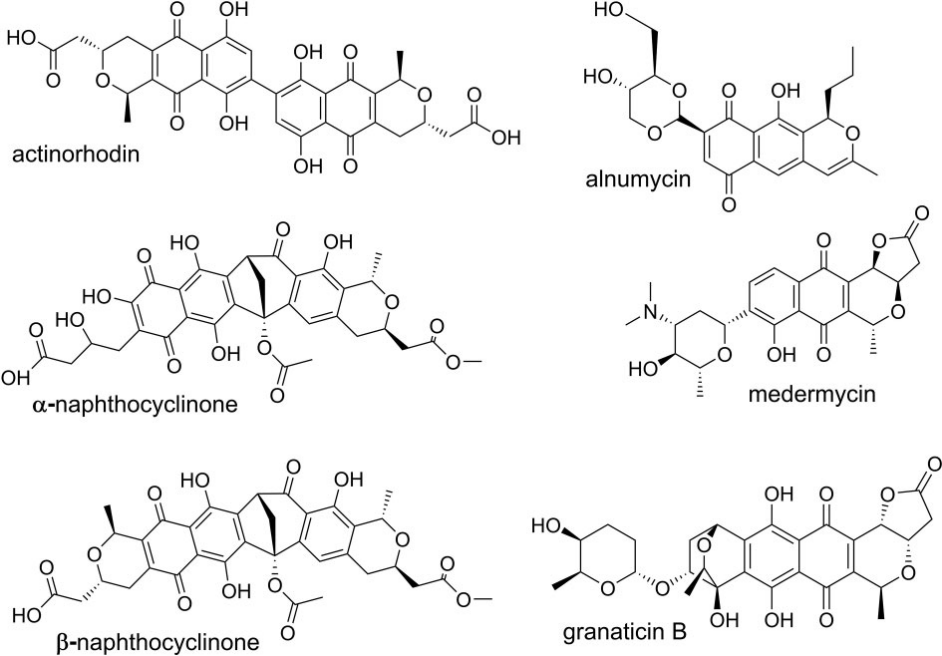
Chemical structures of actinorhodin, alnumycin, granaticin, medermycin, and the α, β-forms of naphthocyclinone.

Aromatic polyketides are a large subgroup of secondary metabolites that harbour a wide range of pharmaceutical functions (Medema et al. 2015), which include the antibacterial tetracycline (Pickens and Tang 2009) and the anticancer agent doxorubicin (Hulst et al. 2021). One extensively studied type-II polyketide is the benzoisochromanequinone (BIQ) antibiotic actinorhodin synthesized by the model organism *Streptomyces coelicolor* A3(2) (Okamoto et al. 2009; Taguchi et al. 2013; Hashimoto et al. 2023). Other notable examples of BIQ polyketide antibiotics include naphthocyclinones, alnumycin, granaticin and medermycin (Metsä-Ketelä et al. 2013) (Fig. 1). Naphthocyclinones pertinent to this study were originally discovered in 1974 from *Streptomyces arenae* DSM 40737 (Zeeck and Mardin 1974, Zeeck et al. 1974) and subsequently confirmed to be asymmetrical dimers (Krone et al. 1982, Ando et al. 2015). Three different conformations of naphthocyclinones, the α, β-, and γ-forms, have been identified, but only the β- and γ-forms show bioactivities against Gram-positive bacteria (Brünker et al. 2001). One notable difference between actinorhodin and naphthocyclinone is the mode of dimerization, since the two monomers are joined together either by one or two C-C bonds, respectively.

Early ^13^C labelling experiments have indicated that both actinorhodin and naphthocyclinone are formed via dimerization of two 16-carbon polyketides (Schröder and Floss 1978, Gorst-Allman et al. 1981). The molecular genetics of actinorhodin biosynthesis have been studied since the 1970s (Rudd and Hopwood 1979). The carbon chain is synthesized from eight malonyl-CoA units via iterative Claisen condensations by the ketosynthase α (KSα)/KSβ heterodimer. The highly reactive polyketide is tethered to the acyl carrier protein (ACP) to prevent aberrant cyclization events. The polyketide is folded to a bicyclic intermediate that is released from the ACP by a distinct 9-ketoreductase (9-KR), first ring aromatase/cyclase (ARO/CYC) and second ring cyclase (CYC). Next 3-ketoreduction (3-KR) promotes pyran ring cyclization and the monomer unit of actinorhodin, dihydrokalafungin, is formed by a set of redox enzymes (Metsä-Ketelä et al. 2013). The atypical short-chain dehydrogenase/reductase (SDR) ActVA-4 has been implicated in the dimerization reaction, since inactivation of the gene resulted in the accumulation of 8-hydroxy-dihydrolalafungin (Taguchi et al. 2012).

In contrast, the biosynthesis of naphthocyclinone has received considerably less attention to date. Earlier work identified two DNA fragments from the biosynthetic gene cluster (BGC) (Sherman et al. 1989, Brünker et al. 1999, 2001) and very recently the genes were identified from the genome sequence of *S. eurocidicus* CGMCC 4.108 (Li et al. 2023). Here we have sequenced the genome of *S. arenae* DSM 40737 and captured the BGC for heterologous production of naphthocyclinone in *S. albus* J1074. We provide experimental evidence that *ncnN* and *act*VA-3 are involved in the dimerization of naphthocyclinone and actinorhodin, respectively.

## Materials and methods

### Strains, oligonucleotides and chemicals

*Streptomyces arenae* DSM 40737 was used as the source of the *ncn* BGC, while *Streptomyces albus* J1074 was used as a host for heterologous expression studies. *Streptomyces coelicolor* M145 was used as a host to study actinorhodin biosynthesis. Cloning vectors and *Escherichia coli* cell lines (Supporting information text) were obtained kindly from Prof. A. Francis Stewart, Genomics, Biotechnology Center, Technische Universität Dresden, Germany. Oligonucleotide primers used in our experiment were purchased from Eurofins Genomics GmbH Germany. All the chemicals, endonucleases, and reagents used in our experiment were purchased from Merck, USA, unless otherwise stated.

### Genome analysis

Genomic DNA was extracted as previously reported (Nikodinovic et al. 2003). Sequencing was done at Eurofins Genomics (Ebersberg, Germany) using Illumina MiSeq v3 (2 × 300 bp). Read quality was checked using FASTQC (v0.11.2) (Andrews 2015). Assembly was performed using A5-MiSeq (v20150522)(Coil, Jospin and Darling 2015). Contiguation was performed with ABACAS (v1.3.1) (Assefa et al. 2009) using *S. albus* NK660 (CP007574.1) as a reference and the gaps were filled using IMAGE (v2.4.1)(Tsai, Otto and Berriman 2010). The assembly was annotated using RAST (Brettin et al. 2015). All programs were run on the CSC—IT Center for Science's Taito super-cluster (Espoo, Finland). Finally, the assembly was run on antiSMASH (v6.0.1) (Blin et al. 2019) for biosynthetic gene cluster prediction and on BiG-FAM (Kautsar et al. 2021) to uncover novel BGCs. This Whole Genome Shotgun project has been deposited at DDBJ/ENA/GenBank under the accession JAKQYH000000000. The version described in this paper is version JAKQYH010000000. The naphthocyclinone biosynthetic gene cluster was deposited in the Minimum Information about a Biosynthetic Gene cluster (Medema et al. 2015) (MIBiG) database under the accession number BGC0000248. The biosynthetic gene clusters were visually compared using EasyFig (Sullivan, Petty, Beatson 2011).

### General DNA techniques and gene cloning

The strategy for cloning and recombineering of the *ncn* BGC was carried out as devised by Wang et al. (2016) with minor modifications as described in the Supporting information text. The inactivation of *act*VA-3 was carried out by homologous recombination using vector pWHM3 (Vara et al. 1989), including an additional oriT sequence to allow conjugation to Streptomyces, as described in the Supporting information text.

### Production and purification of α-naphthocyclinone acid

The *S. albus*/SA-naphtho cells were grown in 4 L NoS-soyE1, which is modified E1 without starch (Ylihonko et al. 1994, Oja et al. 2008) at 30^○^C in 50 mL batches for 7 d and 300 mL batches for 8 d with an agitation of 250 rpm in both cases. The cells were harvested and discarded. The supernatant was subjected to ethyl acetate extraction with 1% (v/v) of acetic acid. The extraction was repeated 2–4 times. The ethyl acetate fractions were combined and dried. Fractions were stored at −20^○^C. Part of the sample was solubilized in chloroform and loaded to the column and the part that did not solubilize was dissolved in acetone and dried with silica and dry-loaded to the column. The silica columns were equilibrated with 10:90 acetone:chloroform. A gradient from 10 to 100% of acetone was run, but the main compound did not elute from the column. Finally, 100% methanol was used to elute the main compound, which was collected and dried. The dried sample was solubilized with methanol and subjected to preparative HPLC, LC-20 AP with a diode array detector SPD-M20A (Shimadzu) with a C-18 column (Kinetex Prep C18, 5 µm, 250 × 21.2 mm; Phenomenex). The fractions containing the main compound were combined and extracted with chloroform, dried, and stored at −20^○^C. The yield of α-naphthocyclinone acid was 2 mg per liter culture.

### Production and purification of fogacin

The *S. albus*/SA-naphtho/ΔncnN cells were grown in E1 soy media (glucose: 20 g/L; soy powder: 5 g/L; yeast-extract: 2.5 g/L; K_2_HPO_4_.3H_2_0: 1.3 g/L; MgSO_4_.3H_2_O: 1 g/L; NaCl: 3 g/L; CaCO_3_: 3 g/L; pH: 7.5) at 30^○^C with an agitation of 250 rpm for 8 days. For this, a seed culture was prepared a day before the main culture, and 10% of it was inoculated to the main production culture. To the knockout mutants, Apra (50 µg/mL) was added to the growth media. After 8 d, the growth culture was centrifuged to obtain the supernatant, and the cells were discarded. The supernatant was incubated with an adsorbent (LXA1180, Sunresin SEPLITE®, 20 g/L) overnight. The LXA1180 was collected, washed with water and the bound compounds were extracted with 20% (v/v) methanol. The aqueous methanol was acidified with 1% (v/v) acetic acid and extracted with chloroform. The chloroform phase was dried and stored at −20^○^C. The dried sample was dissolved in chloroform and loaded to a silica column equilibrated with 10:90 methanol:chloroform. A gradient from 10% to 100% of methanol was run. The fractions containing the compound of interest were combined and dried. The sample was dissolved in methanol and purified using preparative HPLC using LC-20 AP with a diode array detector SPD-M20A (Shimadzu) with a C-18 column (Kinetex Prep C18, 5 µm, 250 × 21.2 mm; Phenomenex). A mobile phase gradient from 15% acetonitrile including 0.1% formic acid to 100% acetonitrile was used. The sample was extracted with chloroform and dried. The yield of fogacin was 425 mg per liter culture.

### Production of actinorhodin

Spores (1 × 10^7^) of the *S. coelicolor* M145 wild type and knock-out mutant *S. coelicolor* M145Δ*act*VA-3 were used to inoculate 8 cm plate containing 25 mL of R5 solid medium (consisting of 10 g/L glucose, 5 g/L yeast extract (Difco^TM^), 0.1 g/L casamino acids, 0.25 g/L K_2_SO_4_, 10.12 g/L MgCl_2_.6H_2_O, 5.73 g/L TES buffer, 2 ml/L trace element solution, 20 g/L agar (pH 7.2)) at 30^○^C for 3 days. The culture was homogenized and metabolites were extracted with ethyl acetate. Actinorhodin production was calculated based on area percentage at 520 nm.

### Analysis of metabolites

Shimadzu's SCL-10Avp HPLC with an SPD-M10Avp diode array detector was used to perform analytical HPLC analyses. The analyses to detect naphthocyclinones were performed with a reversed-phase column (Phenomenex, Kinetex, 2.6 µm, 4.6 × 100 mm) using gradients from 15% acetonitrile containing 0.1% formic acid to 100% acetonitrile. Production of actinorhodin was analysed using a reversed-phase column (Phenomenex Luna Phenyl-Hexyl 100, 10-µm, 250- by 10-mm column) using a gradient from 0.1% Trifluoroacetic acid in water to 100% acetonitrile. MS analyses were carried out with either a low-resolution MS with an HPLC system (Agilent 1260 Infinity 6120 Quadropole LC-MS) with similar conditions and column as the analytical HPLC, or with a MicrOTOF-Q high-resolution MS with direct injection (Bruker Daltonics). NMR samples were prepared from overnight desiccated compounds with deuterated methanol (α-naphthocyclinone acid) and deuterated acetone, methanol, and DMSO for fogacin. NMR analysis was performed with a 600 MHz Bruker AVANCE-III NMR-system equipped with liquid nitrogen cooled Prodigy TCI (inverted CryoProbe) at 298–300 K. The experiments included 1D (^1^H, ^13^C) and 2D measurements (COSY, HMBC, HSQC and additionally NOESY for fogacin). Topspin (Bruker Biospin) was used for spectral analysis.

## Results and discussion

### Genome sequencing of *S. arenae* DSM 40737

**Figure 2.**
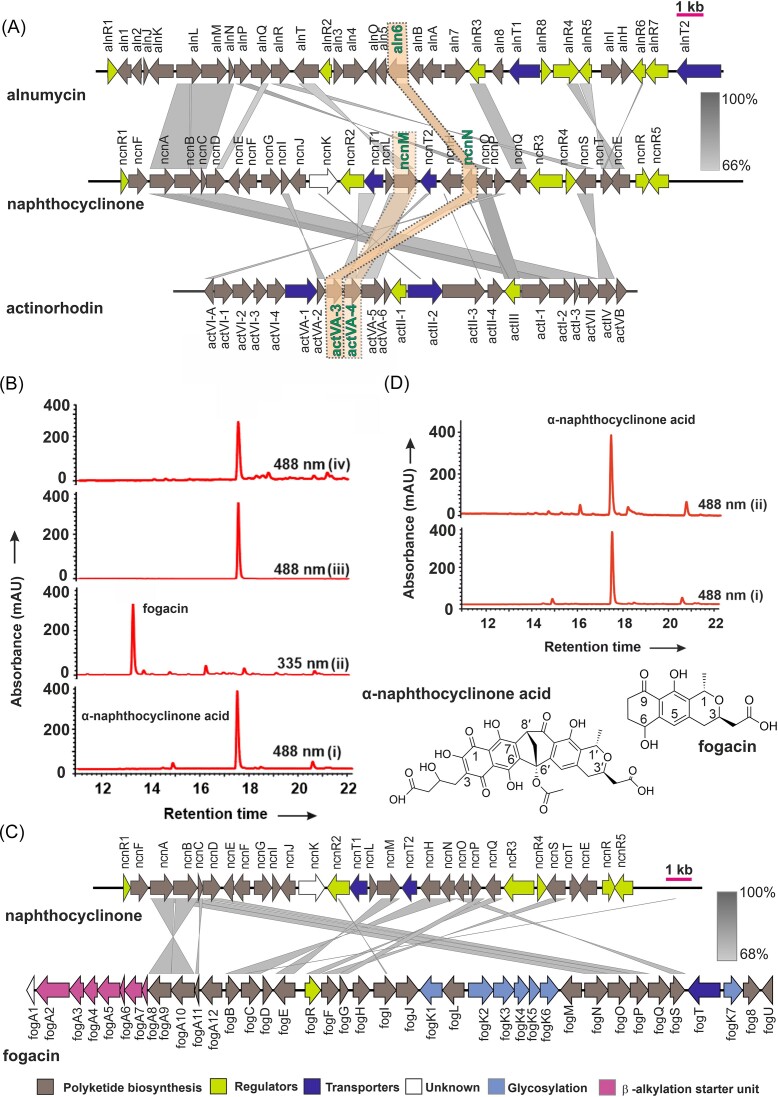
Naphthocyclinone biosynthetic gene cluster and heterologous expression trials. (A) Gene organization of the naphthocyclinone *ncn* biosynthetic cluster and comparison to actinorhodin *act* and alnumycin *aln* BGCs. The genes involved in naphthocyclinone dimerization are highlighted. Genes with significant sequence similarity (66% to 100%) in the different gene clusters are connected with grey arrows. (B) Heterologous expression of the *ncn* BGC in *S. albus* and inactivation of *ncnN*. HPLC chromatogram traces of (i) *S. albus*/pSA-naphtho harbouring the intact *ncn* BGC demonstrates production of α-naphthocyclinone acid, while (ii) inactivation of *ncnN* leads to production of fogacin in *S. albus*/pSA-naphthoΔncnN, (iii) authentic α-naphthocyclinone acid NMR standard and (iv) complementation of the *ncnN* mutation with an intact copy restores α-naphthocyclinone acid production in *S. albus*/pSA-naphthoΔncnN/pEN-SV1-ncnN. Chemical structures of α-naphthocyclinone acid and fogacin. (C) Comparison of the *ncn* BGC to the fogacin *fog* BGC reveals conserved genes for polyketide biosynthesis (brown), while genes involved in regulation (yellow) and transport (dark blue) differ. In addition, the fog BGC harbors genes for glycosylation (light blue) and beta-alkylation (purple). (D) Comparative metabolic profiling reveals that inactivation of *ncnM* does not alter α-naphthocyclinone acid production in (i) *S. albus*/pSA-naphtho and (ii) *S. albus*/pSA-naphthoΔncnM.

We initiated the study by extracting high quality genomic DNA from *S. arenae* DSM 40737 and sequencing the genome using MiSeq resulting in 9560148 reads, which were normalized, error corrected, and trimmed down to 7644475 reads. The final *de novo* draft genome assembly consisted of 111 contigs covering 10.5 Mbp with an N50 of 205 194. Annotation of the genome and analysis by antiSMASH (Blin et al. 2021) revealed 42 BGCs involved in secondary metabolism (Table S1), 27 of which are similar to known clusters. BiG-FAM analysis (Kautsar et al. 2021) identified 36 gene cluster families representing the wide chemical diversity of the strain. Moreover, this strain contains 10 complete BGCs having a distance greater than 900 in BiG-FAM analysis indicating that they may encode novel molecules (Table S1). Finally, we identified three type II polyketide BGCs encoding genes for biosynthesis of naphthocyclinone (Fig. 2A), a putative fluostatin-type compound and spore pigment from the sequencing data.

### Bioinformatic analysis of early steps in naphthocyclinone biosynthesis

**Scheme 1.**
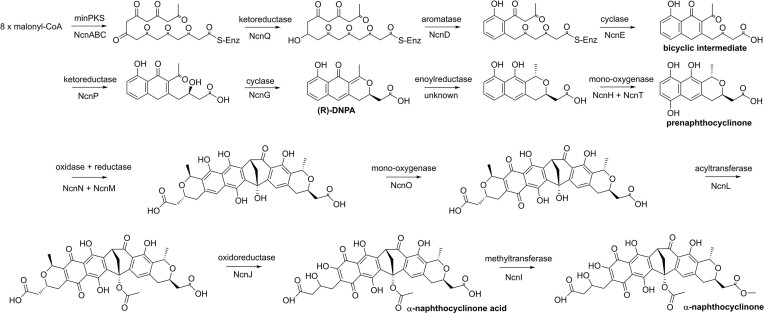
Proposed scheme for the biosynthesis of α-naphthocyclinone acid in *S. arenae*.

Sequence analysis revealed a total of 19 genes that could be responsible for the biosynthesis of naphthocyclinones with eight genes for regulation, self-resistance and transport (Table 1). The biosynthetic logic could be inferred by comparison to related BIQ pathways (Metsä-Ketelä et al. 2013). We propose that naphthocyclinone biosynthesis initiates with the assembly of an octaketide polyketide on the NcnC ACP through condensation of eight malonyl-CoA units by the NcnA/NcnB KSα/KSβ heterodimer (Scheme 1). The ACP-bound polyketide is subsequently modified by the NcnQ 9-KR, the NcnD ARO, and the NcnE CYC to generate the common enzyme-free bicyclic intermediate on BIQ pathways.

**Table 1.** Sequence analysis of genes residing in the naphthocyclinone *ncn* gene cluster of *S. arenae* DSM 40737.

Gene name	Size (AA)	RAST annotation	Most similar ORF [species] (identity %)	Accession no.
*ncnA*	421	Keto-synthase α	Beta-ketoacyl-[acyl-carrier-protein] synthase family protein [*Streptomyces humi*] (99)	WP_071659785.1
*ncnB*	429	Keto-synthase β	Ketosynthase chain-length factor [*Streptomyces humi*] (98)	WP_071659786.1
*ncnC*	86	Acyl carrier protein (ACP)	Acyl carrier protein [*Streptomyces humi*] (100)	WP_046733492.1
*ncnD*	315	Aromatase, AlnQ	Aromatase/cyclase [*Streptomyces humi*] (97)	WP_206306334.1
*ncnE*	310	Cyclase, AlnR/ActIV	MBL fold metallo-hydrolase [*Streptomyces humi*] (97)	WP_206306335.1
*ncnF*	314	Peptidase	M56 family metallopeptidase [*Streptomyces humi*] (95)	WP_046730330.1
*ncnG*	186	Hypothetical protein (possibly assist in pyran ring formation like ActVI-3)	Hypothetical protein [*Streptomyces humi*] (94)	WP_052768782.1
*ncnH*	405	FMN dependent mono-oxygenase	Acyl-CoA dehydrogenase family protein [*Streptomyces humi*] (92)	WP_079179093.1
*ncnI*	287	O-methyltransferase	SAM-dependent methyltransferase [*Streptomyces humi*] (99)	WP_079179091.1
*ncnJ*	314	F420 dependent luciferase like mono-oxygenase	TIGR03619 family F420-dependent LLM class oxidoreductase [*Streptomyces humi*] (99)	WP_052768781.1
*ncnK*	143	Hypothetical protein	PPOX class F420-dependent oxidoreductase [*Streptomyces humi*] (99)	WP_206306330.1
*ncnL*	401	Acyl transferase mdmB	Acyltransferase [*Streptomyces humi*] (98)	WP_046734700.1
*ncnM*	292	Homologous to ActVA-4 (Dimerization)	NmrA/HSCARG family protein [*Streptomyces humi*] (98)	WP_046734699.1
*ncnN*	180	ActVA-3/Aln6 (Dimerization)	DUF6081 family protein [*Streptomyces humi*] (88)	WP_143145067.1
*ncnO*	344	FMN mono-oxygenase (Gra29)	LLM class flavin-dependent oxidoreductase [*Streptomyces humi*] (98)	WP_046734701.1
*ncnP*	245	Keto-reductase	SDR family oxidoreductase [*Streptomyces humi*] (98)	WP_046734697.1
*ncnQ*	261	Keto-reductase (AlnP)	3-oxoacyl-ACP reductase FabG [*Streptomyces humi*] (98)	WP_071659787.1
*ncnS*	510	Unknown protein (Possible role in generation of malonyl-CoA	Acyl-CoA carboxylase subunit-beta [*Streptomyces humi*] (99)	WP_079179094.1
*ncnT*	170	Flavin reductase (AlnH)	Flavin reductase family protein [*Streptomyces humi*] (99)	WP_046732231.1
*ncnR*	202	MerR family (AlnR6/Gra-orf10)	Response regulator transcription factor [*Streptomyces humi*] (99)	WP_046732232.1
*ncnR1*	127	Transcriptional regulator, MecI family	BlaI/MecI/CopY family transcriptional regulator [*Streptomyces humi*] (98)	WP_046730331.1
*ncnR2*	264	Transcriptional regulator, TetR family	TetR family transcriptional regulator [*Streptomyces humi*] (95)	WP_063777123.1
*ncnR3*	142	Hypothetical protein	Nuclear transport factor 2 family protein [*Streptomyces humi*] (99)	WP_046732229.1
*ncnR4*	269	Transcriptional regulator, SARP family	AfsR/SARP family transcriptional regulator [*Streptomyces humi*] (98)	WP_046732230.1
*ncnR5*	430	Putative two-component system sensor kinase	Histidine kinase [*Streptomyces* sp. CBMA123] (56)	WP_188304378.1
*ncnT1*	498	Multidrug resistance protein B	DHA2 family efflux MFS transporter permease subunit [*Streptomyces humi*] (99)	WP_052768780.1
*ncnT2*	136	Hypothetical protein	DoxX family protein [*Streptomyces humi*] (99)	WP_046734698.1

Pyran ring formation is preceded by 3-ketoreduction, which determines the stereochemical outcome of cyclization. The naphthocyclinone pathway is likely to follow the paradigm established for granaticin biosynthesis, since the pathways harbours the SDR enzyme NcnP that is homologous to the 3-KR Gra-6 (Ichinose et al. 1998). The equivalent step in actinorhodin biosynthesis is catalysed by a protein of the 3-hydroxyacyl-CoA dehydrogenase family (Metsä-Ketelä et al. 2013) for which no homologous enzymes are encoded by the naphthocyclinone BGC. The NcnG CYC is the likely candidate for pyran ring cyclization and formation of (*R*)-DNPA (4-dihydro-9-hydroxy-1-methyl-10-oxo-3-*H*-naphtho[2,3-c]pyran-3-acetic acid) (Scheme 1). An interesting deviation from canonical BIQ pathways is that the naphthocyclinone BGC does not contain any gene products of the NAD(P)H dependent medium-chain alcohol dehydrogenases that are typically associated with enoyl reduction and therefore the gene for this step is unknown (Metsä-Ketelä et al. 2013). However, quinone formation is likely to be catalysed by the two-component mono-oxygenases NcnH/NcnT that are homologous to experimentally characterized proteins from the actinorhodin (Valton et al. 2006; Hashimoto et al. 2020) and alnumycin (Grocholski et al. 2012) systems.

### Insight into naphthocyclinone dimerization

In order to obtain experimental evidence for genes involved in the dimerization of naphthocyclinone, we cloned the BGC using *Rec*ET direct cloning (Wang et al. 2016) to facilitate recombineering efforts (Fig. S1). The main metabolite (Fig. 2B) produced by the generated strain *S. albus*/pSA-naphtho was found to be α-naphthocyclinone acid (Zeeck and Mardin 1974). The compound was isolated from the culture broth with acidic ethyl acetate and purified further by silica column and preparative HPLC using a reversed phase column. The structure was verified by NMR (Figs S2–S7, Table S2) and MS analysis (ESI *m/z* [M-H]^−^ obs. 651.1353 calc. 651.1355), which correlated to previously published data (Zeeck and Mardin 1974).

Sequence analysis revealed two candidate gene products for dimerization. NcnM (Table 1, Fig. S8) is a member of the NmrA family of NAD(P)H dependent reductases and homologous to ActVA-4, which is responsible for dimerization in actinorhodin biosynthesis (Taguchi et al. 2012). In addition, we considered that NcnN (Table 1, Fig. S9) could be involved in the reaction, since it shares sequence identity to the co-factor independent oxidase Aln6 from the alnumycin pathway that catalyses C-C bond cleavage (Oja et al. 2008, 2012).

We proceeded to disrupt *ncnN* by recombineering in *E. coli* and conjugated the resulting plasmid pSA-naphthoΔncnN to *S. albus*. Analysis of culture extracts (Fig. 2B) revealed loss of α-naphthocyclinone acid production and accumulation of another metabolite, which was identified as the octaketide fogacin based on NMR (Figs S10–S16, Table S3) and MS (ESI *m/z* [M-H]^−^ obs. 305.1019, calc. 305.1031) analyses. Fogacin has previously been isolated from *S. lividans* (Santamaría et al. 2018), *Streptomyces* sp. Tü 6319 (Radzom et al. 2006) and *S. violaceoruber* (Lu et al. 2014). The relative stereochemistry was verified to be *trans* by NOESY (Figs S15 and S16), where H-3 was coupled with the methyl group H-13, but not with H-1. Complementation of *ncnN* with an intact copy in strain *S. albus*/SA-naphthoΔncnN/pEN-SV1-ncnN restored production of α-naphthocyclinone acid (Fig. 2B). A fogacin *fog* BGC has recently been identified from *Actinoplanes missouriensis* (Sato et al. 2019) and comparison to the *ncn* BGC revealed a homologous gene set for polyketide biosynthesis (Fig. 2C). However, significant differences were also apparent, since the *ncn* BGC contains additional tailoring genes for dimerization and further modification (see below), while the *fog* BGC harbors genes for production of the glycosylated fogacin B and the β-alkylated fogacin C (Sato et al. 2019).

Next, we used recombineering to inactivate *ncnM* resulting in strain *S. albus*/pSA-naphthoΔncnM. Surprisingly, the gene deletion did not have an effect in the production profile of the strain (Fig. 2D) and α-naphthocyclinone acid (Fig. 2D) was detected as the main secondary metabolite. These results are in contrast to experiments on the actinorhodin pathway, where the homologous *act*VA-4 has been shown to be essential for dimerization (Taguchi et al. 2012). We surmise that the result may be due to endogenous complementation of the *ncnM* deletion by the *S. albus* J1074 host strain, which contains an uncharacterized SDR gene (accession number WP_033240217.1) with 40.0% sequence identity to *ncnM*.

### Bioinformatic analysis of late-stage tailoring steps in naphthocyclinone biosynthesis

After dimerization, we propose that the flavoenzyme NcnO would catalyse asymmetrical quinone formation (Scheme 1). Late steps in naphthocyclinone biosynthesis have very recently been investigated in the *nap* BGC from strain *S. eurocidicus* CGMCC 4.108, where experimental data indicates that acylation by NcnL may be the next biosynthetic step (Li et al. 2023). The key difference in the two naphthocyclinone BGCs is that the *ncn* pathway encodes a F_420_ -dependent oxidoreductase NcnJ, for which there is no equivalent gene on the *nap* pathway. We suggest that NcnJ is involved in formation of α-naphthocyclinone, which has not been detected from *S. eurocidicus* CGMCC 4.108, and is responsible for late-stage pyran ring opening and oxidation. The final biosynthetic step is likely to be conversion of α-naphthocyclinone acid to α-naphthocyclinone by the methyltransferase NcnI.

### Mechanism of dimerization in the biosynthesis of benzoisochromanequinone antibiotics

**Figure 3.**
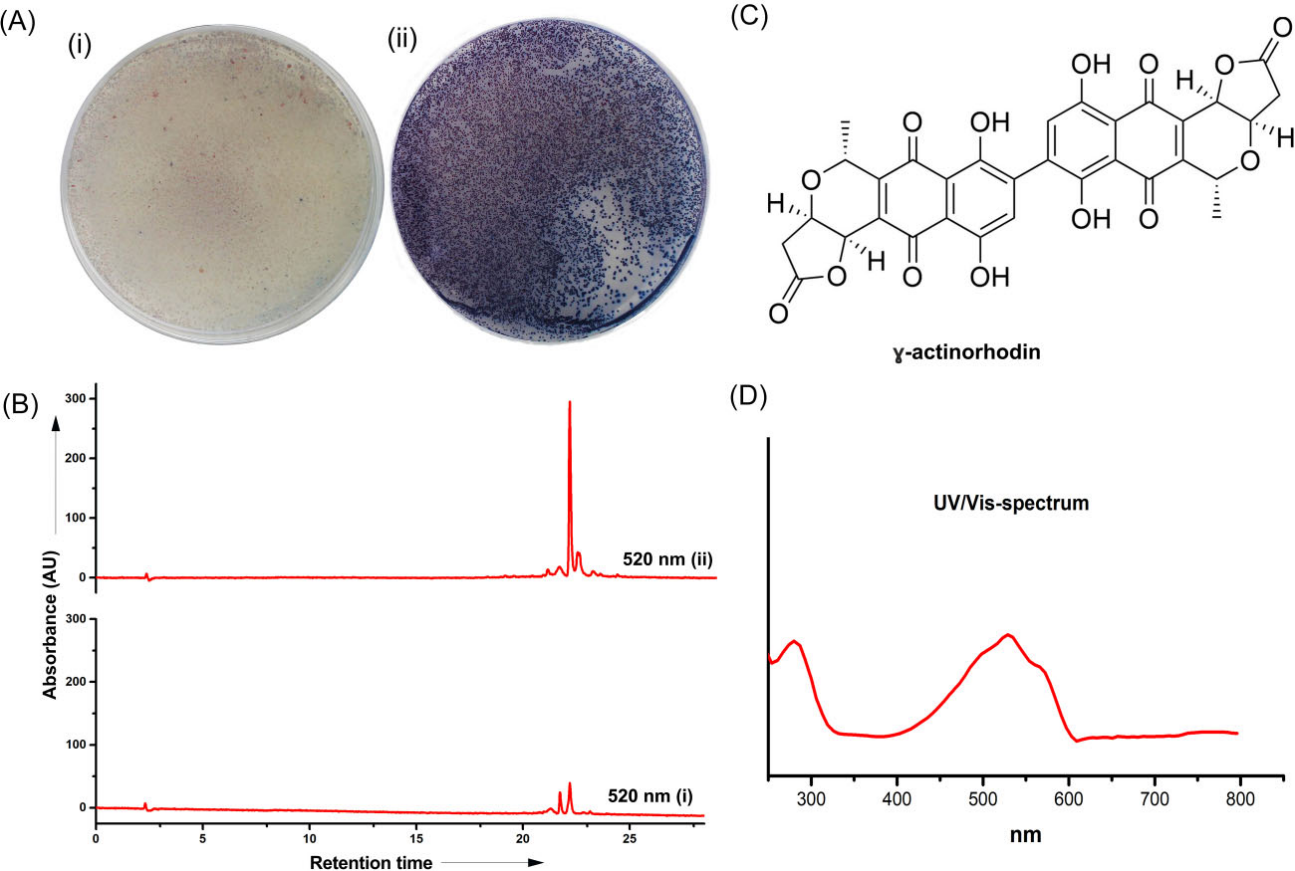
Functional analysis of *act*VA-3. (A) Phenotypic effect of the *act*VA3 deletion on ACT biosynthesis demonstrates reduced production of blue pigmented actinorhodin in the mutant strain in comparison to the wild type. Strains were inoculated on R5 solid medium and grown at 30°C for 3 days. The reverse side of plates is shown for (i) *S. coelicolor* M145ΔactVA-3 mutant and (ii) *S. coelicolor* M145 wild type. (B) HPLC chromatogram traces recorded at 520 nm of culture extracts from *S. coelicolor* M145 wild type (top) and *S. coelicolor* M145ΔactVA-3 mutant (bottom) demonstrates reduced production of actinorhodin in the mutant. (C) Chemical structure of γ actinorhodin and (D) UV/Vis spectrum of γ actinorhodin.

Next, we turned our attention to *act*VA-3 from the actinorhodin pathway, which is homologous to *ncnN* (Fig. 2A), in order to determine if the gene product is required for dimerization along with *act*VA-4 product. We generated a knock-out mutant in *S. coelicolor* M145 based on classical homologous recombination. The target gene *act*VA-3 was replaced by an apramycin- resistance (apr^r^) cassette resulting in strain *S. coelicolor* M145Δ*act*VA-3 (Fig. S18). Comparative metabolic profiling revealed severely impaired actinorhodin production, since the mutant strain accumulated only approximately 8% of actinorhodin in comparison to the wild type (Fig. 3A and B). This result is in agreement with very recent data from the Ichinose laboratory, where the authors demonstrated similar reduction in actinorhodin biosynthesis in the *act*VA-3 mutant and *in vitro* dimerization activity with ActVA-3 and ActVA-4 (Hashimoto et al. 2023). LC-MS analysis was carried out to analyse the mass spectrum of the metabolite. The metabolite displayed the *m/z* value at 629.0 in a negative mode, agreeing to data for authentic γ actinorhodin (Fig. 3C and D; Fig. S18C).

**Scheme 2.**
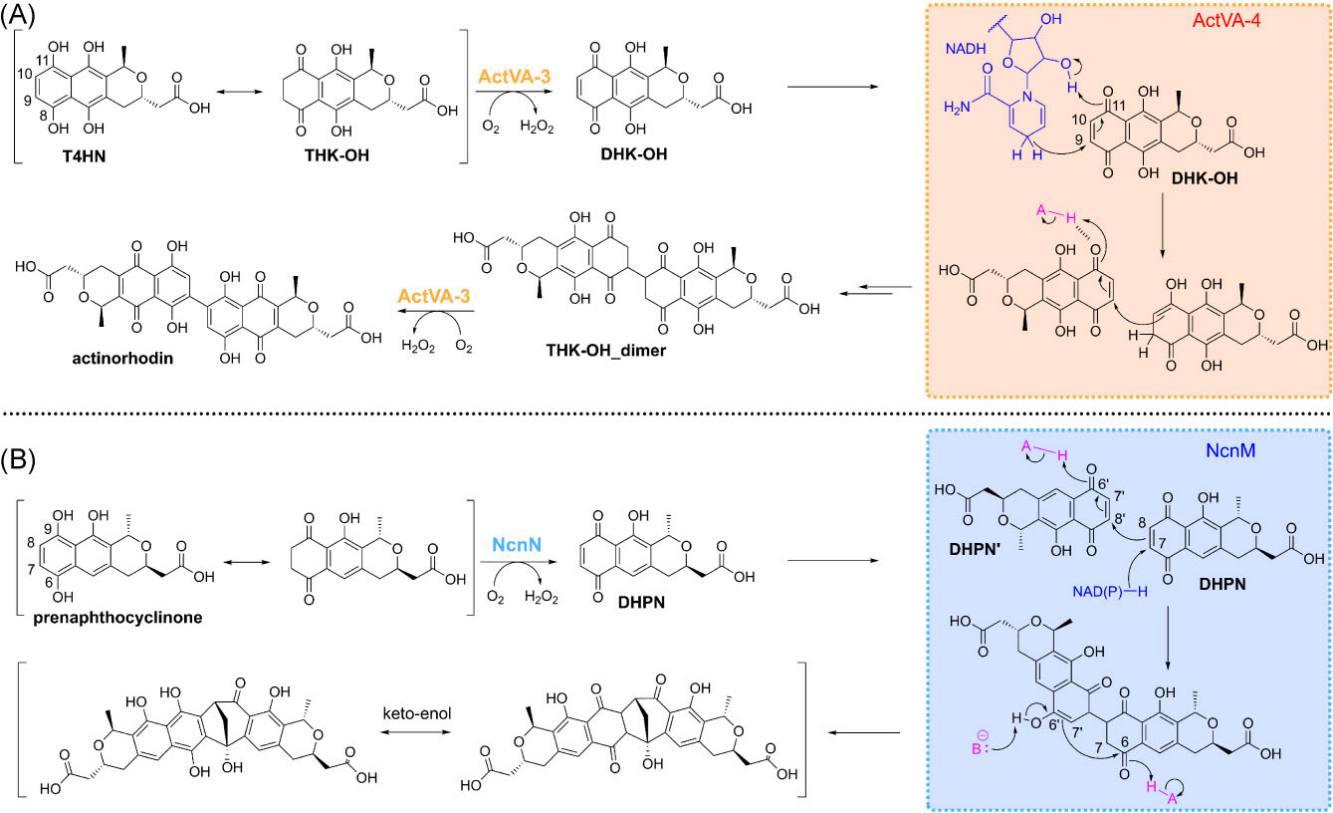
Proposed mechanism for dimerization in actinorhodin and naphthocyclinone pathways.

Our data is in agreement with the recently proposed mechanism for dimerization on the actinorhodin pathway (Hashimoto et al. 2023) (Scheme 2A), where the tetrahydroxynaphthalene product (T4HN) formed by ActVA-5/ActVB, is converted into hydroxytetrahydrokalafungin (THK-OH) through keto-enol tautomerization. ActVA-3 would then use molecular oxygen to oxidize THK-OH into 8-hydroxy-dihydrokalafungin (DHK-OH) by abstracting two adjacent hydrogen atoms and forming hydrogen peroxide in the process. Then ActVA-4 catalyses the dimerization of DHK-OH through a hydride transfer from the NADPH cofactor to the reactive double bond at position 9 of DHK-OH, which together with protonation of the carbonyl at position 11 leads to the formation of an 10/11 enolic intermediate. The π-electrons of the enol then function as a Michael donor attacking the 9’/10’ double bond (Michael acceptor) of another molecule of DHK-OH, forming the THK-OH dimer, which is then oxidized into actinorhodin by ActVA-3 by again abstracting two hydrogens and reducing molecular oxygen into hydrogen peroxide.

We propose that naphthocyclinone dimerization proceeds through a similar mechanism (Scheme 2B). In an orthologous manner to ActVA-3, keto/enol tautomerization of prenaphthocyclinone would allow its oxidation into the corresponding quinone (dehydroprenaphthocyclinone, DHPN) by NcnN using molecular oxygen. The differences in the two pathways would occur in the dimerization reaction, although the initial first step might proceed through similar chemistry. A C-C bond would be formed between the carbon atoms at positions 8 and 8’ of two copies of DHPN through Michael addition. The Michael donor is activated by a hydride transfer from NAD(P)H to the carbon at position 7 of DHPN, which would facilitate adjacent π-electrons to perform a nucleophilic attack on another DHPN molecule (DHPN’), which functions as the Michael acceptor (Scheme 2B). A ketone at position 9 could be involved in resonance stabilization of the formed transient carbanion intermediate, as described for actinorhodin (Scheme 2A). The electrophilicity of the Michael acceptor is increased by a simultaneous protonation of the carbonyl oxygen at position 6’. The unique second step in the naphthocyclinone dimerization would be an aldol reaction between the formed Michael acceptor and the carbonyl carbon at position 6 (Scheme 2B). This reaction would be catalysed by deprotonation of the 6’-enol and protonation of the 6-ketone functional groups. The resulting bicyclic dimer would tautomerize into the corresponding hydroquinone and no additional oxidations by NcnN, in contrast to what has been demonstrated for ActVA-3 in actinorhodin biosynthesis, would be required.

The *S. albus*/pSA-naphthoΔncnN strain produced fogacin, which is likely a shunt product. The diketone tautomer of prenaphthocyclinone, which is normally oxidised by NcnN into DHPN, is instead reduced into fogacin in the absence of NcnN in the heterologous host. Possibly the same enzyme that would normally use NAD(P)H to catalyse the dimerization of DHPN, would catalyse this ketoreduction. Fogacin lacks the necessary 7/8 double bond, and is therefore an unsuitable substrate for dimerization.

## Conclusions

In this study, we have confirmed that *ncnN* and *act*VA-3 are involved in the dimerization of naphthocyclinone and actinorhodin, respectively. Our heterologous expression studies of the naphthocyclinone *ncn* cluster in *S. albus* led to production of α-naphthocyclinone acid. Bioinformatic analysis allowed us to propose a tentative scheme for the generation of α-naphthocyclinone acid. We provide the first insight into the unique asymmetrical naphthocyclinone dimerization via gene inactivation experiments and propose that NcnN is an oxidase that primes naphthocyclinone monomer units for dimerization. Furthermore, our results corroborate recent findings (Hashimoto et al. 2023) that ActVA-3 is directly relevant to actinorhodin biosynthesis in *S. coelicolor*. Deletion of a*ct*VA-3 led to a significant decrease (about 92%) in actinorhodin biosynthesis. NcnN and ActVA-3 belong to a poorly characterized enzyme family that has not been structurally characterized, but members of the family appear to be co-factor independent oxidases that utilize molecular oxygen without metal ions or organic co-factors (Oja et al. 2012). Collectively, our results pave the way for more detailed mechanistic studies in order to understand how NcnN and ActVA-3 are able to break the spin barrier between organic molecules and molecular oxygen possibly in a co-factor independent manner.

## Supplementary Material

fnad123_Supplemental_File
