# Supplementary material for: Co-factor independent oxidases ncnN and actVA-3 are involved in the dimerization of benzoisochromanequinone antibiotics in naphthocyclinone and actinorhodin biosynthesis
Source: FEMS Microbiol Lett. 2023 Nov 21;370:fnad123. doi: 10.1093/femsle/fnad123 (PMC10697411; doi:10.1093/femsle/fnad123)
Supplement: fnad123_Supplemental_File [file fnad123_supplemental_file.docx]

**Supporting Information:**

**Dimerization of benzoisochromanequinone antibiotics in naphthocyclinone and actinorhodin biosynthesis**

Bikash Baral^[1]^, Soheila Matroodi^[1,2]^, Vilja Siitonen^[1]^, Keshav Thapa^[1]^, Amir Akhgari^[1]^, Keith Yamada^[1]^, Aleksi Nuutila^[1]^ and Mikko Metsä-Ketelä^[1,^*^]^

^[1]^ Department of Life Technologies, University of Turku, Finland

^[2]^ Department of Marine Biology, Faculty of Marine Science and Oceanography, Khorramshahr University of Marine Science and Technology, Iran

**Table of Contents**

Supporting information text

Figure S1. BGC capture and digestion by different endonucleases.

Figure S2. Proton spectrum of α-naphthocyclinone acid in CD_3_OD.

Figure S3. Carbon spectrum of α-naphthocyclinone acid in CD_3_OD.

Figure S4. COSY spectrum of α-naphthocyclinone acid in CD_3_OD.

Figure S5. HSQC spectrum of α-naphthocyclinone acid in CD_3_OD.

Figure S6. HMBC spectrum of α-naphthocyclinone acid in CD_3_OD.

Figure S7. HR-MS spectra of α-naphthocyclinone acid.

Figure. S8. Amino-acid sequence alignment of NcnM and ActVA-4.

Figure S9. Sequence comparison between ActVA-3, NcnN and Aln 6.

Figure S10. Proton spectrum of fogacin in CD_3_OD, DMSO-*d6* and acetone-*d6*.

Figure S11. Carbon spectrum of fogacin in CD_3_OD, DMSO-*d6* and acetone-*d6*.

Figure S12. COSY spectrum of fogacin in CD_3_OD, DMSO-*d6* and acetone-*d6*.

Figure S13. HSQC spectrum of fogacin in CD_3_OD, DMSO-*d6* and acetone-*d6*.

Figure S14. HMBC spectrum of fogacin in CD_3_OD, DMSO-*d6* and acetone-*d6*.

Figure S15. NOESY spectrum of fogacin in CD_3_OD, DMSO-*d6* and acetone-*d6*.

Figure S16. COSY and NOESY correlations for fogacin.

Figure S17. HR-MS of fogacin.

Figure S18. Construction of the *S. coelicolor*∆actVA-3 by gene disruption with the apramycin resistance gene (apr^r^). Table S1. Genomic potential of *S. arenae* DSM 40737 for production of secondary metabolites.

Table S2. NMR spectral data of α-naphthocyclinone acid.

Table S3. NMR spectral data of fogacin.

**Supporting information text.**

**Vector selection and amplification of p15A backbone using PCR**: *E. coli* Gbdir-gyrA462 cells harbouring p15A-cm-tetR-tetO-ccdB-hyg plasmid was used for capturing the entire biosynthetic gene cluster of size 30 kb. The oligonucleotides (23 bp that amplifies vector backbone) with 80 bp long homology arms (homologous to the clusters to be cloned) gave rise to 103 bp long oligonucleotides, which were used for the amplification of the vector (p15A-cm-tetR-tetO-ccdB-hyg). The sequences of the two oligonucleotides were: SA_Naph_RecET_F (aaacgtcaggagggcctgcaatatcagggtaaactttccgtagcgctcccgtaacccctgcgcggtgggggcgcaatgtaAGATCCGAAAACCCCAAGTTACG) and SA_Naph_RecET_R (cgctaccgacgtgacgtgccctgacgtgacgtgccctgactgacctgacctgatccctcggaccctctcaccgcaatgtcAGATCCTTTCTCCTCTTTAGATC). The linearized plasmid digested with *Bam*HI was used as a template for the PCR reaction. The 20 µL total PCR reaction volume comprised of GC buffer: 4 µL, dNTPs (10 mM): 0.4 µL, Primer (F and R): 1 µL each, DNA (10 ng), DMSO: 0.6 µL, and DNA polymerase (Phusion: 0.2 µL) in MilliQ water. The thermocycling conditions for PCR reaction were initial denaturation of 95 ^○^C (2 m), followed by 30 cycles of denaturation at 98 ^○^C (10 s), annealing at 58 ^○^C (15 s), elongation at 72 ^○^C (3 min), and a final extension at 72 ^○^C (10 min).

**Preparation of the electrocompetent *E. coli* cells:** *E. coli* cells GB05RedTrfA harbouring pSC101-BAD-ETgA-tet plasmid was used as a cloning vector. The cells were used to inoculate 1.4 mL LB broth medium supplemented with 4 µg/mL tetracycline in an Eppendorf tube to prepare electrocompetent cells at 300 rpm, 30 ^○^C, 2 h. After OD reached 0.35 and 0.4, the induction for the expression of *Rec*E/*Rec*T was made by employing 35 µL of 10% l-arabinose. The induced cells were shifted to 37 ^○^C for 40 minutes. The cells were harvested when OD_600_ reached between 0.7-0.8 (7,700 × *g*; 30 s; 2 ^○^C). The pellet was washed thoroughly with autoclaved MilliQ water and again resuspended in 1 mL of MilliQ water. The process was repeated with centrifugation at 9,400 × *g* and 11,400 × *g* each for 30 s at 2 ^○^C. Finally, the cell pellet was resuspended in MilliQ water (20 µL) and was used for electroporation. For electroporation, freshly prepared electrocompetent cells were used.

**Genomic DNA (gDNA) digestion:** The 27,128 bp long naphthocyclinone biosynthetic gene cluster from *S. arenae* could be cut out with a single endonuclease, namely *Bsr*DI, from the genome of *S.* *arenae*. The 400 µL master-mix for the digestion of gDNA includes: 10× digestion buffer (40 µL), gDNA (67 µL; 200 ng/µL), RNase A (2 µL; 10 µg/µL), endonuclease (16 µL), MilliQ (275 µL). The mixture was incubated for complete digestion (37 ^○^C; 4 h). Following the digestion of gDNA, the digested DNA fragments were extracted using phenol-chloroform-isoamyl (25:24:1). After centrifugation (9,400 × *g*; 10 min), the DNA in the water phase was precipitated with 20 µL of 3M sodium acetate and 800 µL of absolute ethanol, which was then washed with 70% ethanol and dried in a speed vacuum. Following the drying, DNA was resuspended in 12 µL of autoclaved MilliQ, and its concentration was measured with Nanodrop 2000 Spectrophotometer (ThermoFisher Scientific). The digested genomic DNA was used further for electroporation experiments.

**Electroporation of digested gDNA (dgDNA), PCR amplified homology arms of p15A vector, and *E. coli* GB05RedTrfA cells that harbour pSC101-BAD-ETgA-tet plasmid:** The *E. coli* GB05RedTrfA / pSC101-BAD-ETgA-tet plasmid was used as a host for electroporation. The dgDNA (10 µg; 5 µL) from *S. arenae* and 5 µL (1 µg) of amplified PCR products equipped with homology arms (linearized p15A vector with *Bam*HI used as a template) were added to the electrocompetent cells (20 µL). The amplified PCR product with homology arms (verified with sequencing) was used to capture the entire *ncn* pathway, generating plasmid SA-Naph/p15A of size *ca* 31.1 kbp (Fig S1a). Following electroporation (1,350 V, 10 µF, 600 Ω), 1 mL of LB was pipetted into a cuvette to resuspend the cells. This entire mixture was incubated at 37 ^○^C (300 rpm; 1 h). The cells were harvested by spinning them at 6,000 × *g* for 4 min at RT. The supernatant was discarded, and the pellets were resuspended in 100 µL of LB medium. The entire mixture was then plated on LA plates supplemented with 15 µg/mL of CAM. The plates were left to dry and were incubated (37 ^○^C; overnight or until the colonies became visible).

**Isolation and analysis of the plasmid DNA from *E. coli* GBO5 / pSC101-BAD-ETgA-tet cells:** Clone candidates containing the naphthocyclinone BGC in SA-Naph/p15A were picked from LA plates supplemented with 15 µg/mL of CAM and inoculated into 2 mL of LB media supplemented with 10 µg/mL of CAM, followed by overnight incubation at 37 ^○^C with continuous stirring (320 rpm). DNA extraction was performed by alkaline lysis method as per protocol (Sambrook et al., 1989), with some minor changes. The extracted clone candidates were screened using different endonucleases (*Bam*HI, *Bgl*II, *Eco*RI, and *Xba*I) to identify correct clones (Fig S1b). For further confirmation, the sequencing of the plasmid SA-Naph/p15A along with homology arms for capturing the whole pathway was performed.

**LCHR recombination activity:** In order to be able to conjugate the naphthocyclinone BGC to *Streptomyces*, further linear-to-circular homologous recombination (LCHR) was done to include the oriT-attP-phiC31cassette in the DNA construct. For this, the cloned *ncn* pathway in SA-Naph/p15A was cloned to a sub-cloning suicidal vector (pR6K-OriT-tnpA-kan) through LCHR activity. This suicidal vector released a conjugation-transposition cassette to modify a cloning vector. The electro-competent cells *E. coli* GBO5 / pSC101-BAD-ETgA-tet were grown in 1.4 mL of LB media and 35 µL of 10% l-rhamnose was used to induce the expression of Redα/Redβ/Redγ/RecA for LCHR. These cells were used for LCHR events between SA-Naph/p15A and pR6K-OriT-tnpA-kan. Following electroporation and incubation overnight, the obtained clones were checked with digestion by an endonuclease (*Bam*HI) for the presence of the cloned pathway (Fig S1c). The verified construct (5 µL; 500 ng) comprising the pathway (*ncn* pathway) was used for further cloning in the destination vector (oriT-attP-phiC31). The destination vector included integrase (Phic31), and its recombination target site *attP*, which facilitated site-specific integration into the *Streptomyces* genome. The entire mixture was then electroporated into *E. coli* GB05RedTrfA cells, followed by plating on LB agar plates supplemented with 20 µg/mL Apra for oriT-attP-phiC31 insertion. The dried plates were incubated at 37 ^○^C overnight. Following day, extraction, and verification of cosmids from overnight grown culture was performed. The cosmid (*E. coli* GB05RedTrfA/SA-naphtho) was verified with digestion by different endonucleases and the correct cosmid was used to conjugate the expression host *Streptomyces albus* (referred to as *S. albus*/SA-naphtho) in the subsequent text.

**Recombineering:** Within the naphthocyclinone pathway (*S. albus*/SA-naphtho), the genes namely *ncnN* and *ncnM* were targeted for disruption. For disruption, oligonucleotide primers with 50 bp of the desired gene and 20 bp of the desired resistance genes were designed and were purchased in string form from Genewiz (Leipzig, Germany). These primers were used for the PCR amplification of a CAM-resistance (CAM^R^) from the plasmid pKD3. The 50 µL total PCR reaction volume comprised of MilliQ (27.5 µL); GC buffer (10×): 10 µL; DNA template (pKD3): 1 µL (10 ng); Primers (Forward and Reverse): 1 µL (10 pM) each; dNTP (10 mM): 1 µL; DNA polymerase: 1 µL; and DMSO: 1.5 µL. The thermocycling conditions for PCR reaction comprised of initial denaturation of 95 ^○^C (7 min), followed by 35 cycles of denaturation at 94 ^○^C (15 s), annealing at 50 ^○^C (30 s), elongation at 72 ^○^C (90 s), and a final extension at 72 ^○^C (7 min). For gene disruption, *E. coli* GB05RedTrfA/SA-naphtho was made electrocompetent, followed by an insertion of pKD46 plasmid with a CAM-resistance (CAM^R^) gene through electroporation (1250 V, 25 µF). The resistant transformants were selected by plating them on LA plates supplemented with Amp (100 µg/mL) and Apra (50 µg/mL), followed by incubation at 30 ^○^C for 16 h. These resistant clones denoted GB05RedTrfA/SA-naphtho/pKD46 were induced by l-arabinose and made electrocompetent. For this, 50 mL of culture was used which was induced with 380 µL of 20% l-arabinose at OD_600_ 0.1. The cells were harvested when the OD_600_ was 0.8, after which they were made electrocompetent and transformed with PCR-product (100 ng). The transformants were plated on LA plates supplemented with CAM (35 µg/mL), Apra (50 µg/mL) and grown at 37 ^○^C for 16 h. Plasmid extraction and subsequent *Xba*I restriction test were performed for the transformants. This gave rise to product of size 1 kb, confirming the presence of the CAM-resistance gene in place of either *ncnN* or *ncnM*. In the meanwhile, the K12/pFLP2 cells that harbour Amp^R^ were made electrocompetent. These cells were grown at 30 ^○^C and were heat induced by growing at 37 ^○^C when their growth reached OD_600_ 0.17. The electroporation of the gene cluster (SA-naphtho/pKD46) harbouring the CAM^R^ was done in K12/pFLP2 electrocompetent cells, followed by their recovery by plating them on LA plates with Apra (50 µg/mL) at 37 ^○^C for 16 hr. The clones were checked in the secondary plates (LA with Apra+CAM and LA plates supplemented with Apra; same concentration as before) to confirm the loss of the CAM^R^ gene. The CAM-sensitive clones (CAM^S^) were used for the further downstream processes. Plasmid isolation for the CAM^S^ clones was performed by growing them in LB broth overnight at 37 ^○^C. The plasmid extract was analysed by digesting it with an endonuclease (*Xba*I). The absence of a PCR product of size 1 kb confirmed that the CAM resistance was gone. The plasmids were cloned into ET12567/pUZ8002 methyl defective strains and were conjugated to the surrogate host *S. albus* J1074, resulting in *S. albus*/SA-naphthoΔncnN and *S. albus*/SA-naphthoΔncnM. The conjugation was done as described by Kieser et al., (2000) with slight modifications.

**Gene complementation:** The complementation of the disrupted *ncnN* gene was done by cloning an intact copy purchased from Genewiz (Leipzig, Germany) and obtained in a plasmid (pUC-GW-ncnN) downstream of a constitutive promoter SP44. The obtained gene segment was ligated to pEN-SV1 shuttle vector by digesting with *Hin*dIII and *Xba*I, followed by transformation into the knock-out mutants (*S. albus*/ΔncnN) through conjugation. The spreading of streptomycin (50 µg/mL) and nalidixic acid (20 µg/mL) was performed after 18 hours, followed by incubation at 30 ^○^C until the appearance of exconjugants. The exconjugants obtained were streaked for a few generations to obtain single colonies, and the ex-conjugants’ metabolic profiling was performed. The analysis of the production profile of the complementation mutants showed that the production of α-naphthocyclinoic acid was restored.

**Inactivation of actVA-3:** To establish the *in vivo* function of *actVA*-3, we constructed a mutant strain directly in *S. coelicolor* M145 via classical homologous recombination (Kieser et al., 2000). A synthetic DNA fragment (Genewiz, Germany) encoding for an apramycin- resistance (Apr^r^) gene flanked with two 1 kbp homology arms was cloned in *E. coli* into a modified pWHM3 (Vara et al., 1989) vector, with an additional oriT sequence to allow conjugation into Streptomyces, to generate plasmid pWHM3_oriT_*S coeli*_VA3_BB. The plasmid was passed via *E. coli* ET12567/pUZ8002 grown in LB at 37 ^○^C with appropriate antibiotics (100 μg/mL ampicillin, 50 μg/mL thiostrepton, 50 μg/mL apramycin) and conjugated into *S. coelicolor* M145. Thiostrepton sensitive and apramycin resistant clones, indicating a double recombination event, were identified and the resulting strain was designated as *S. coelicolor* M145∆actVA-3. Gene disruption was confirmed by PCR (Figure S18) using specific PCR primers actVA3_F(GACCAAACTTCCCGCTCA), actVA3_R(GCGCTCTACCAGGACT) and apr_R (GCCCATCCATTTGCCT).

**Supplementary Figures**


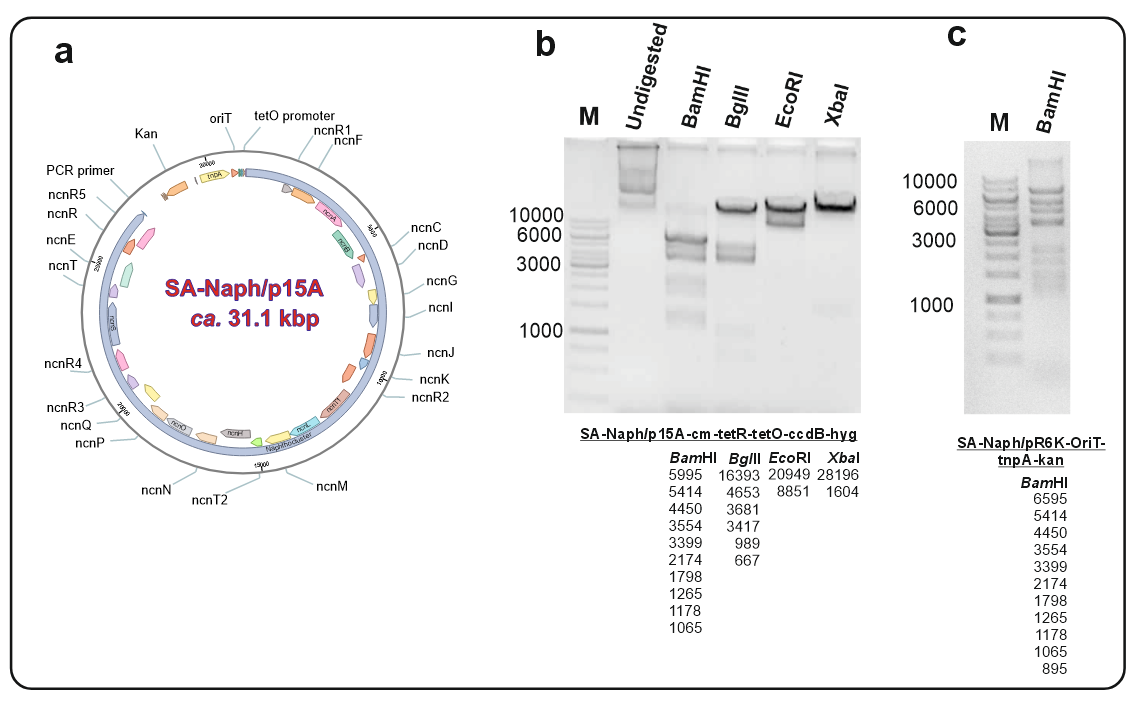


**Figure S1. BGC capture and digestion by different endonucleases.** a) *ncn* BGC cloned in p15A plasmid, b and c) digestion of the cosmid with different endonucleases.


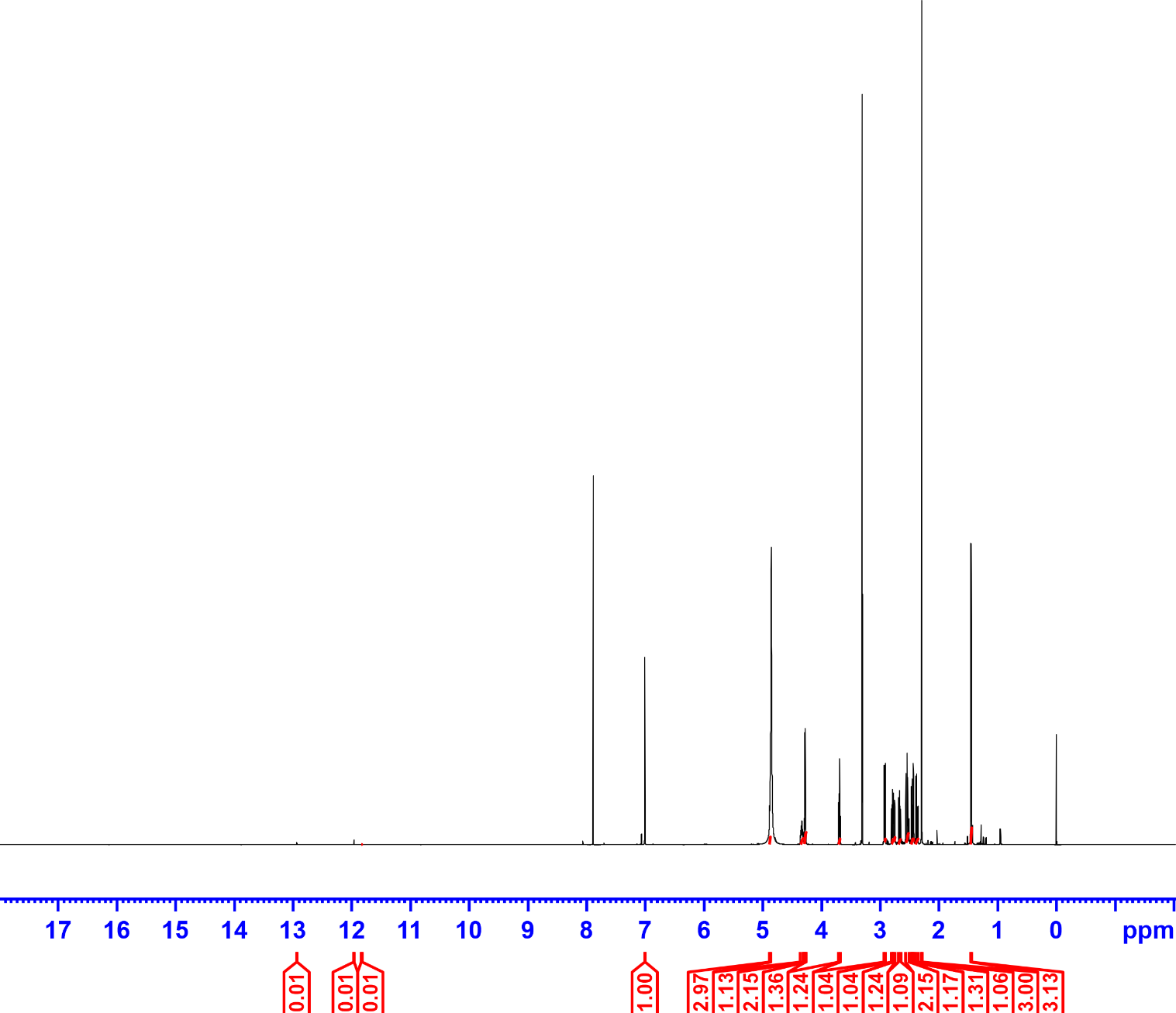


**Figure S2. Proton spectrum of α-naphthocyclinone acid in CD_3_OD.**

**
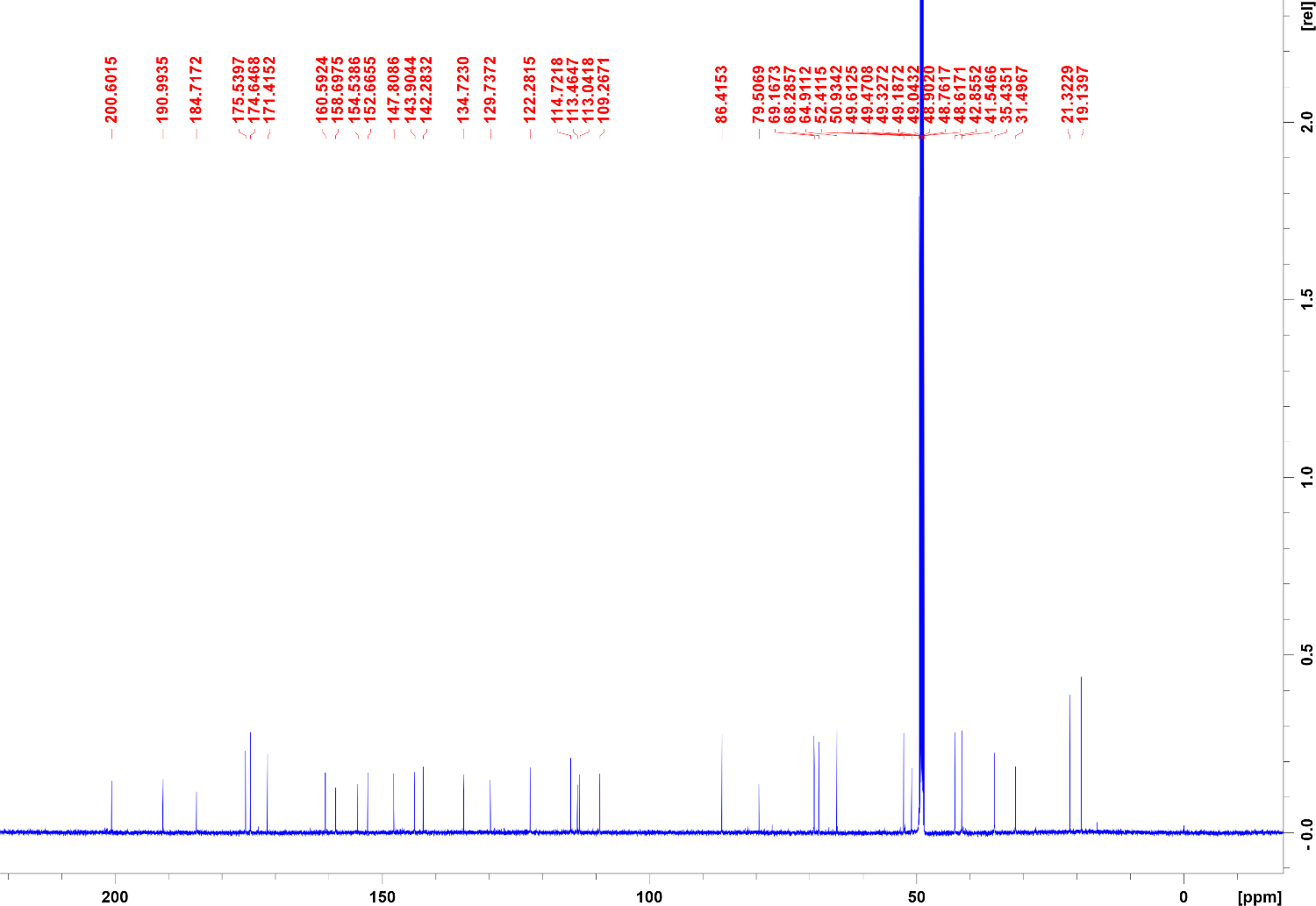
**

**Figure S3. Carbon spectrum of α-naphthocyclinone acid in CD_3_OD.**

**
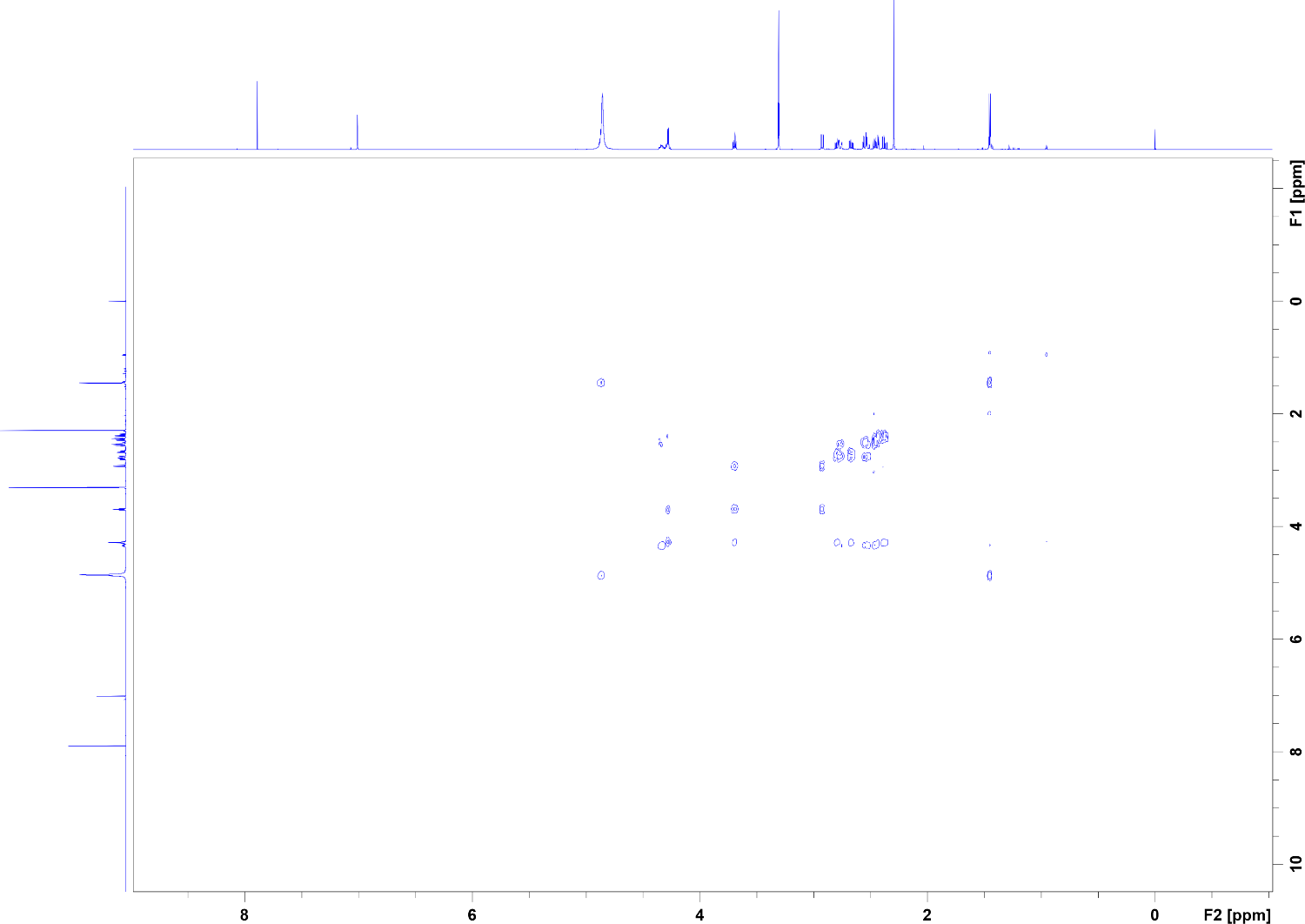
**

**Figure S4. COSY spectrum of α-naphthocyclinone acid in CD_3_OD.**

**
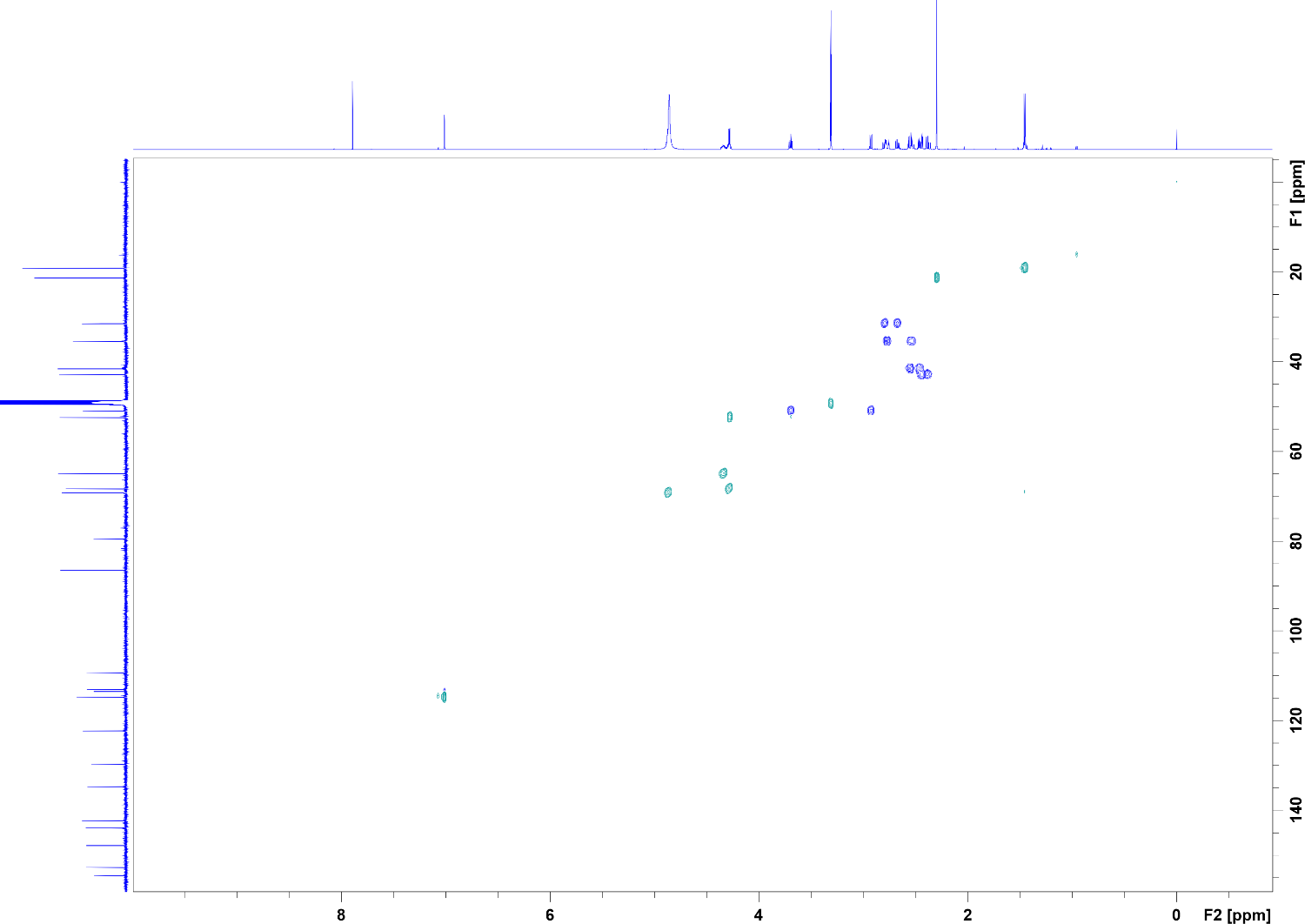
**

**Figure S5. HSQC spectrum of α-naphthocyclinone acid in CD_3_OD.**

**
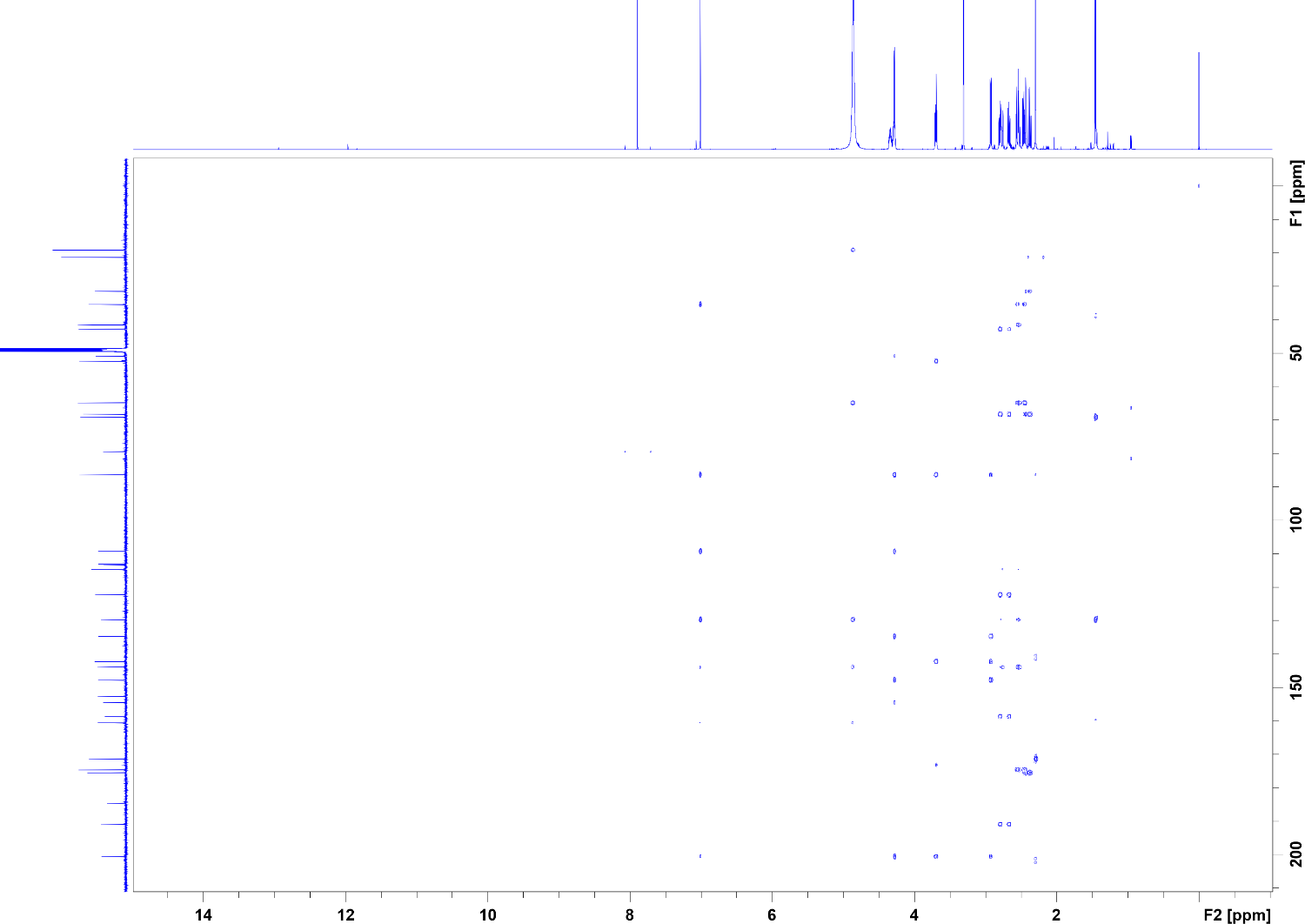
**

**Figure S6. HMBC spectrum of α-naphthocyclinone acid in CD_3_OD.**

**
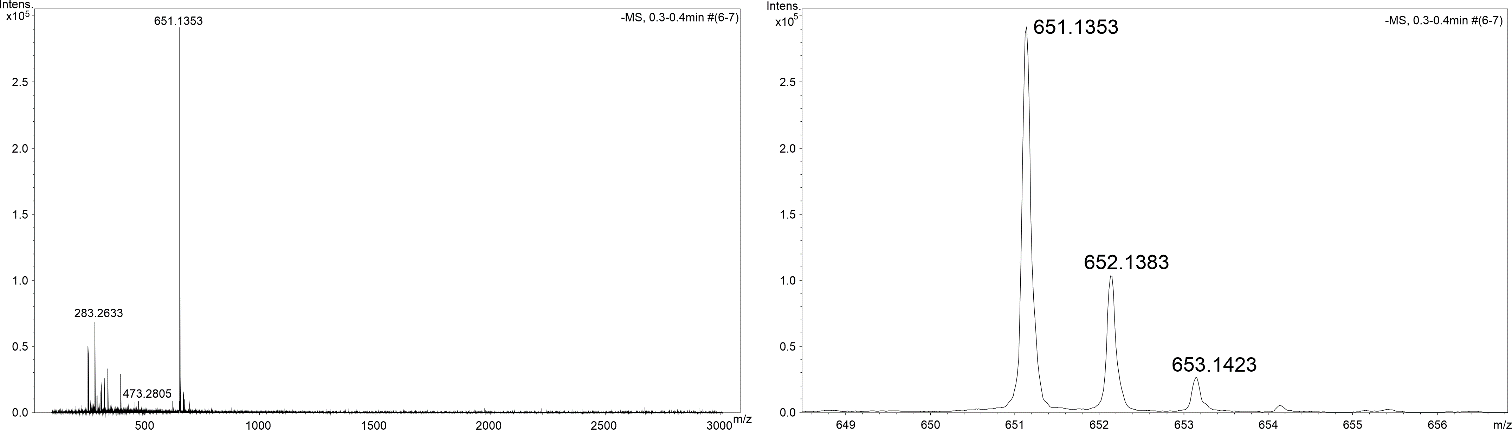
**

**Figure S7. HR-MS spectrum of α-naphthocyclinone acid.** ESI m/z [M-H]-, ESI- obs. 651.1353, calc. 651.1355.


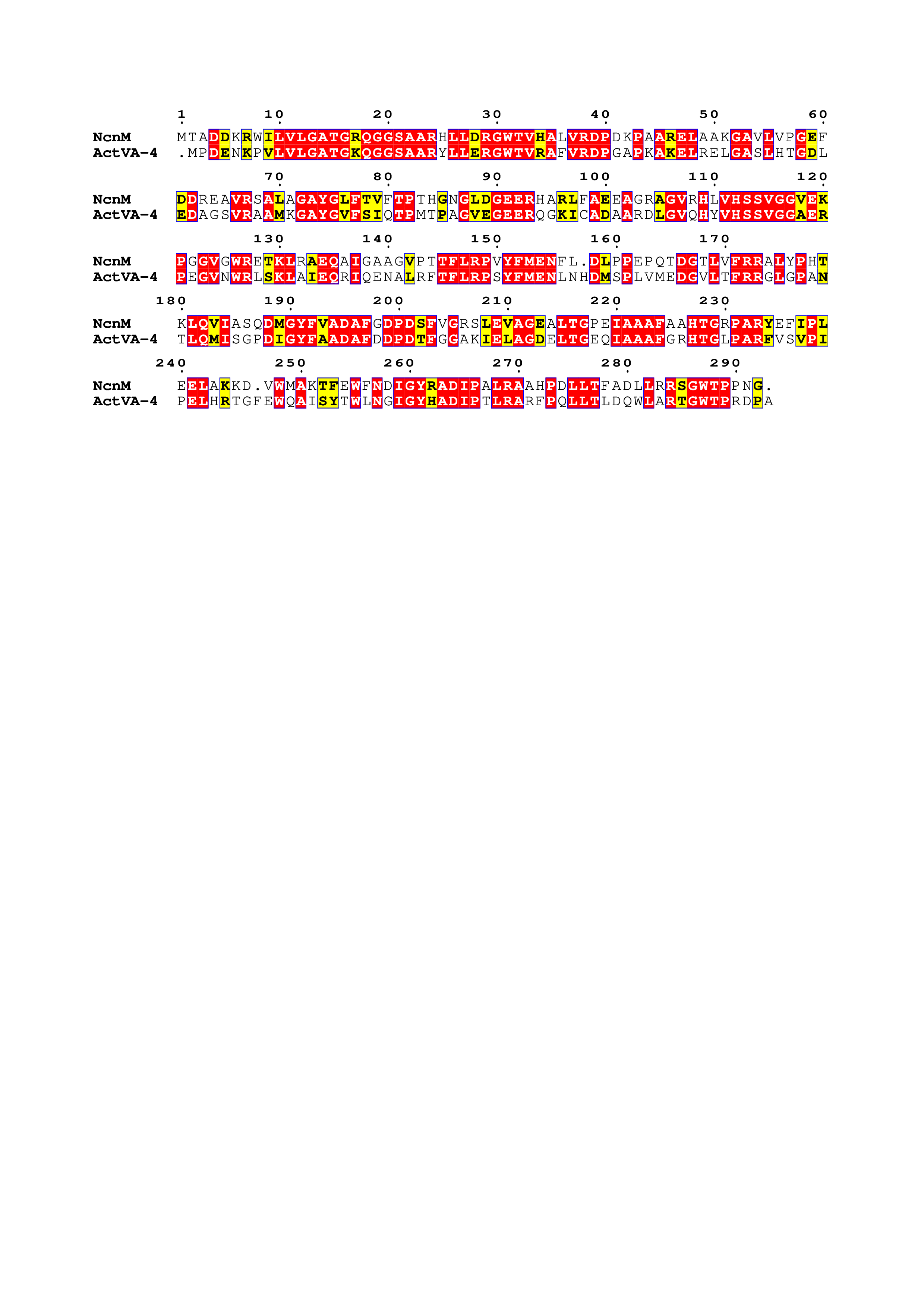


**Figure. S8. Amino-acid sequence alignment of NcnM and ActVA-4.** Alignment was performed using Clustal Omega, while visualization was performed by Espript 3.0.

**
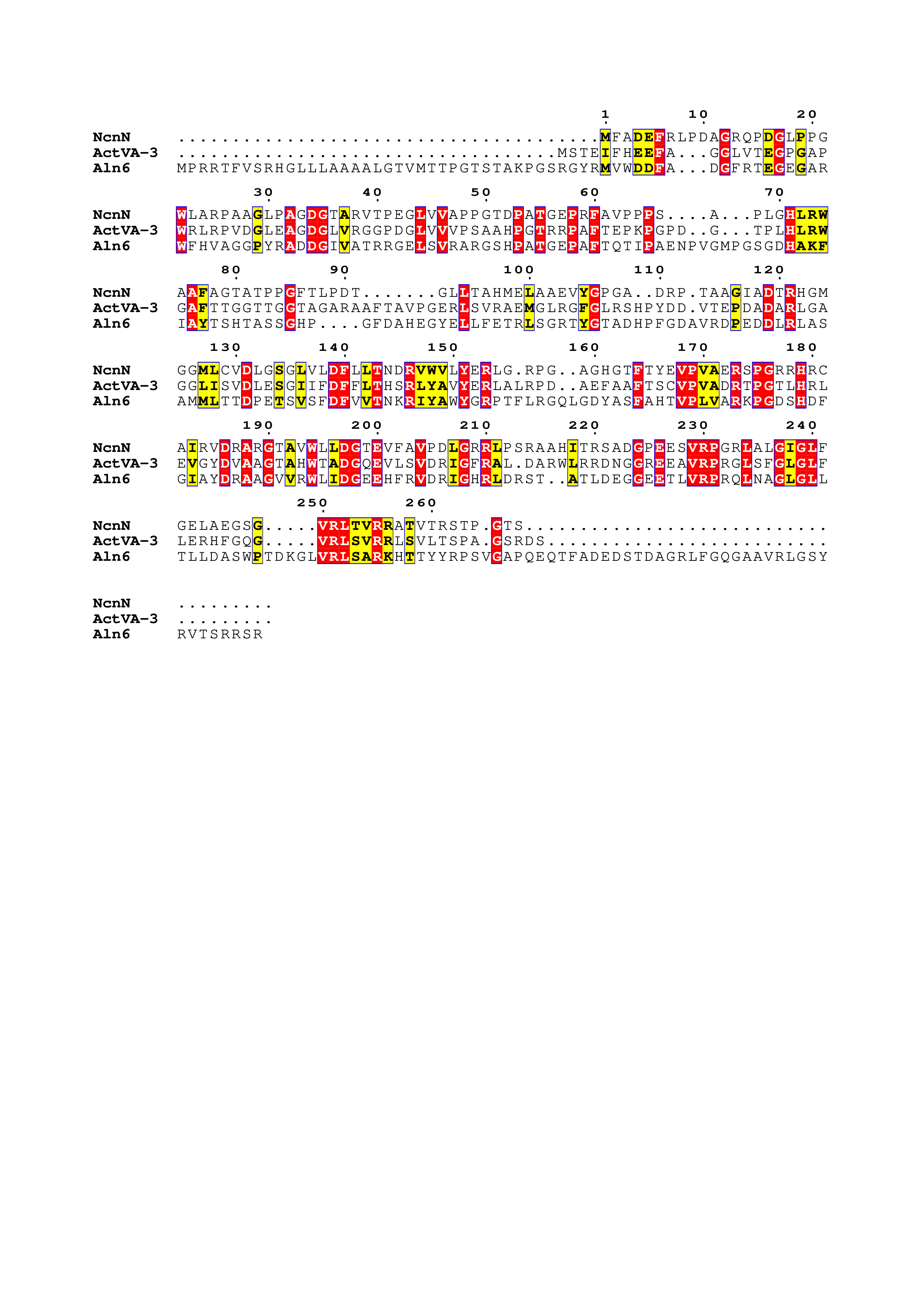
**

**Figure S9. Sequence comparison between ActVA-3, NcnN and Aln 6.** Alignment was performed using Clustal Omega, while visualization was performed by Espript 3.0.

**
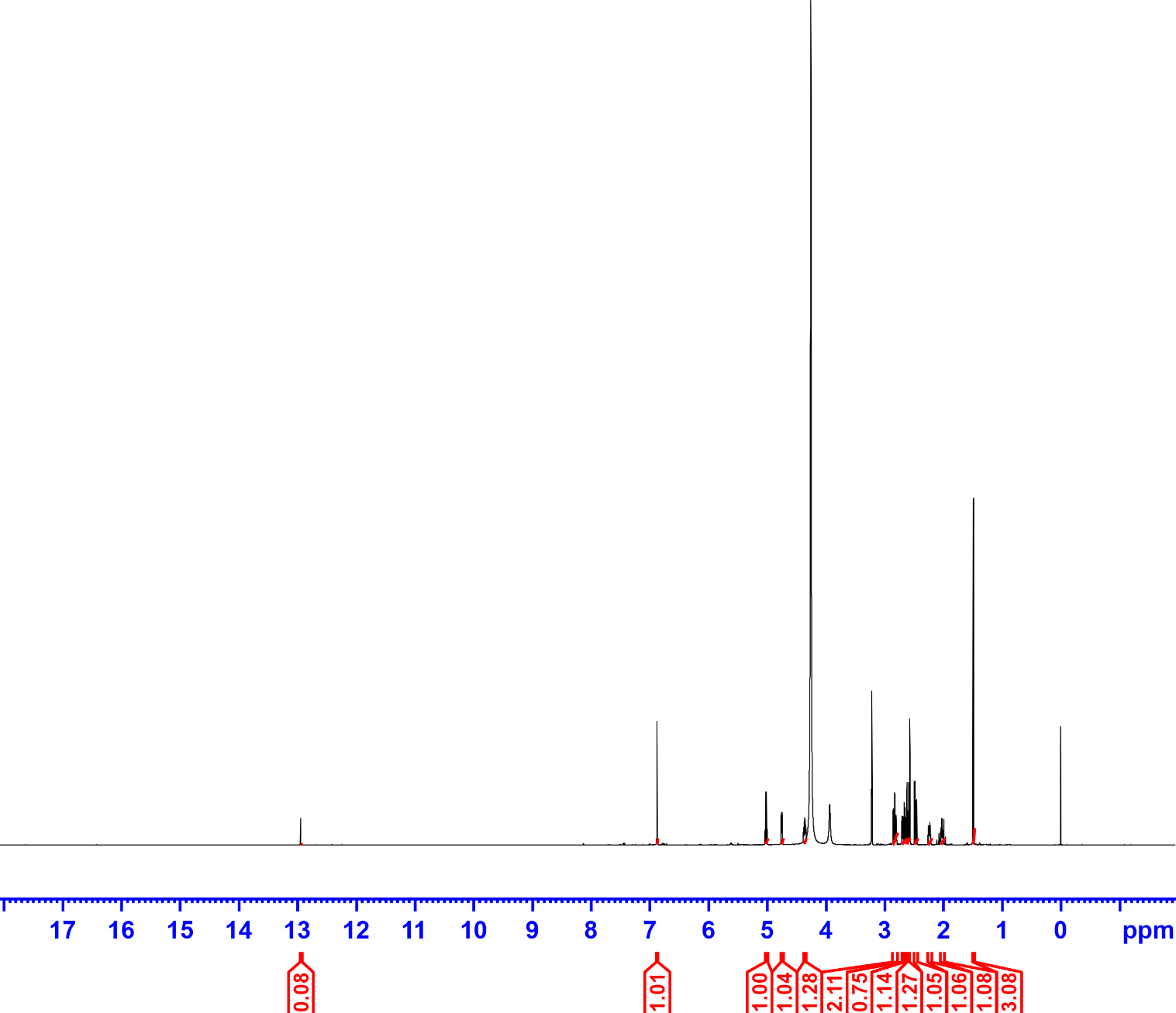
**

**Figure S10. Proton spectrum of fogacin in CD_3_OD, DMSO-*d6* and acetone-*d6*.**

**
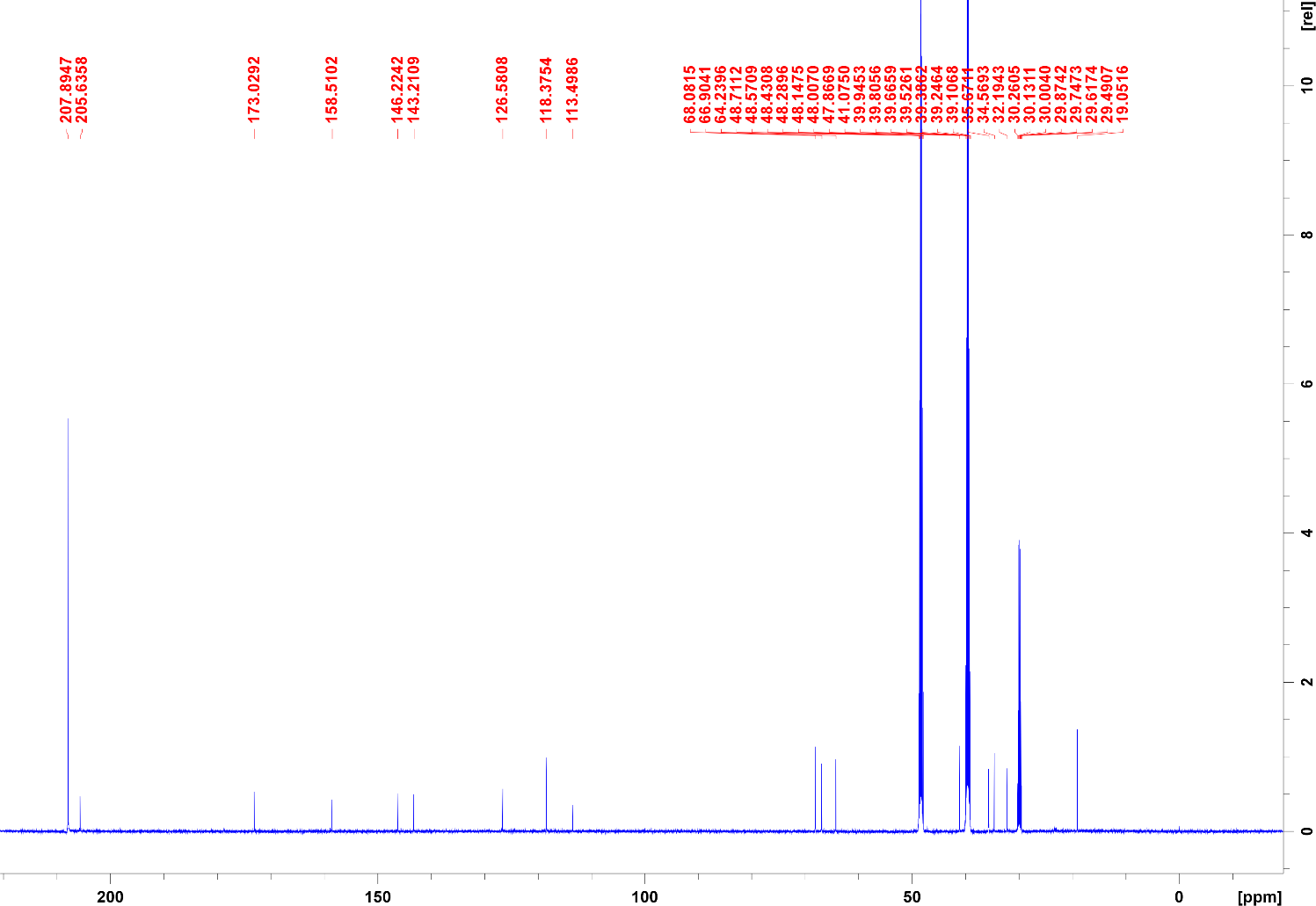
**

**Figure S11. Carbon spectrum of fogacin in CD_3_OD, DMSO-*d6* and acetone-*d6*.**

**
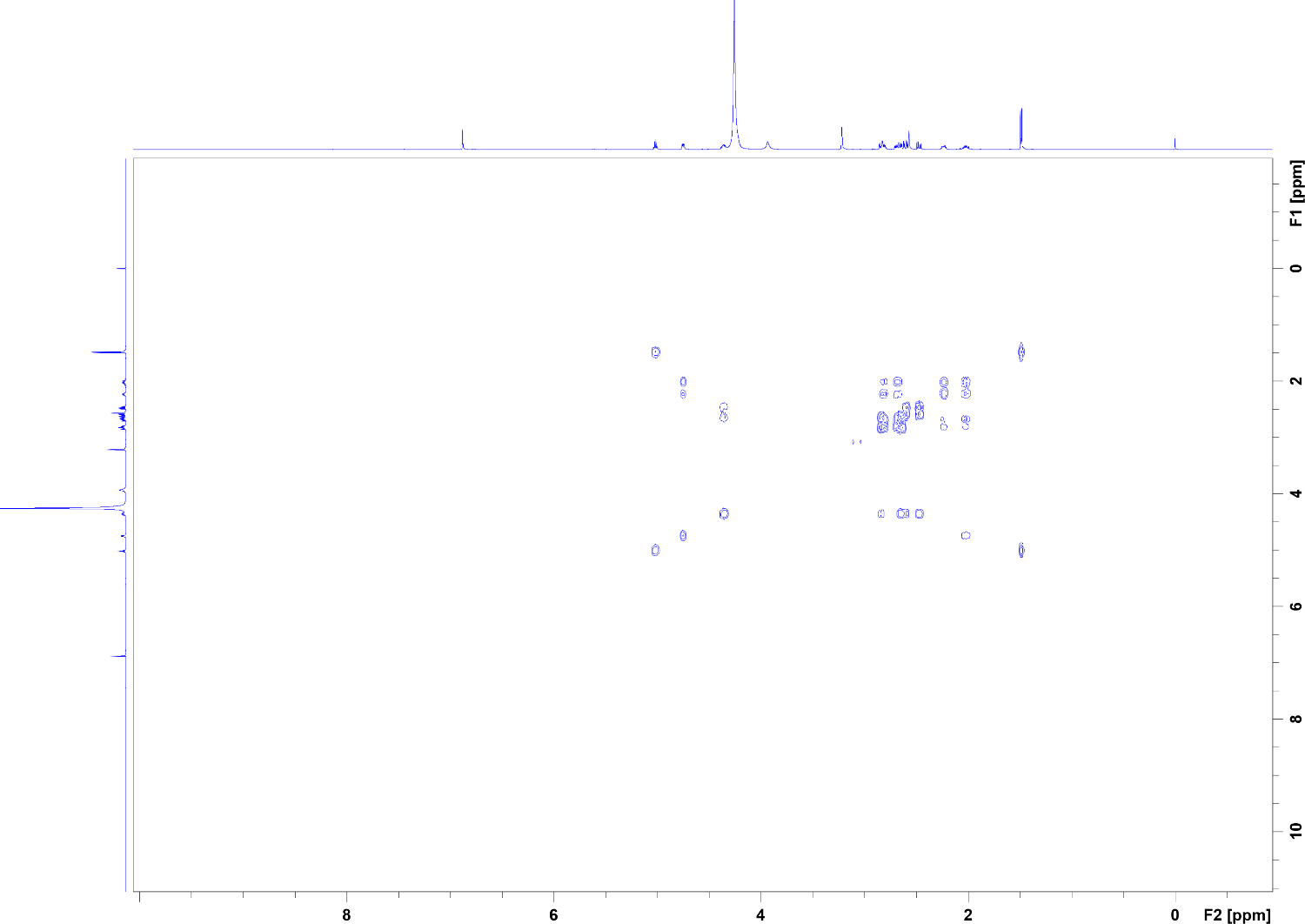
**

**Figure S12. COSY spectrum of fogacin in CD_3_OD, DMSO-*d6* and acetone-*d6*.**

**
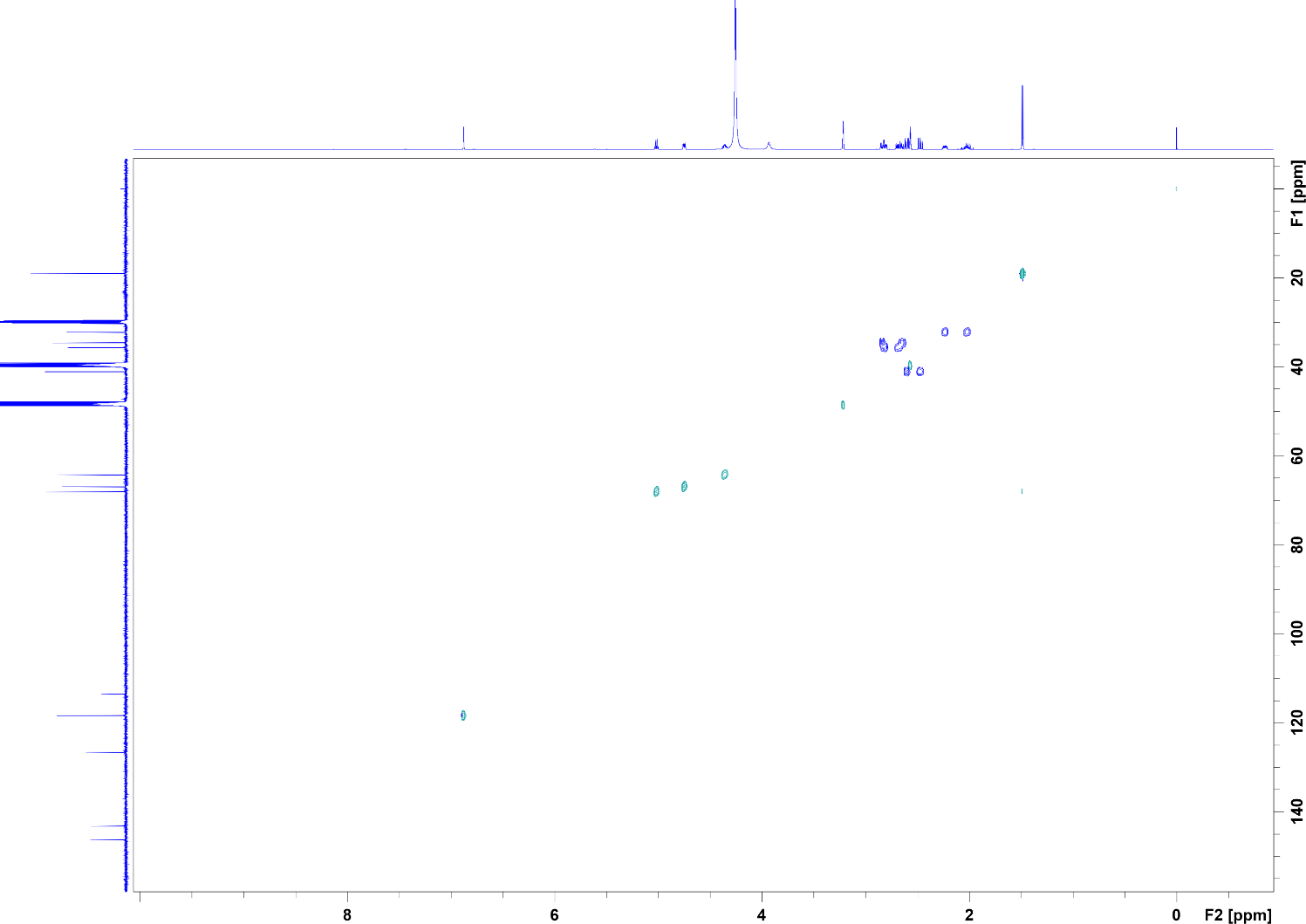
**

**Figure S13. HSQC spectrum of fogacin in CD_3_OD, DMSO-*d6* and acetone-*d6*.**

**
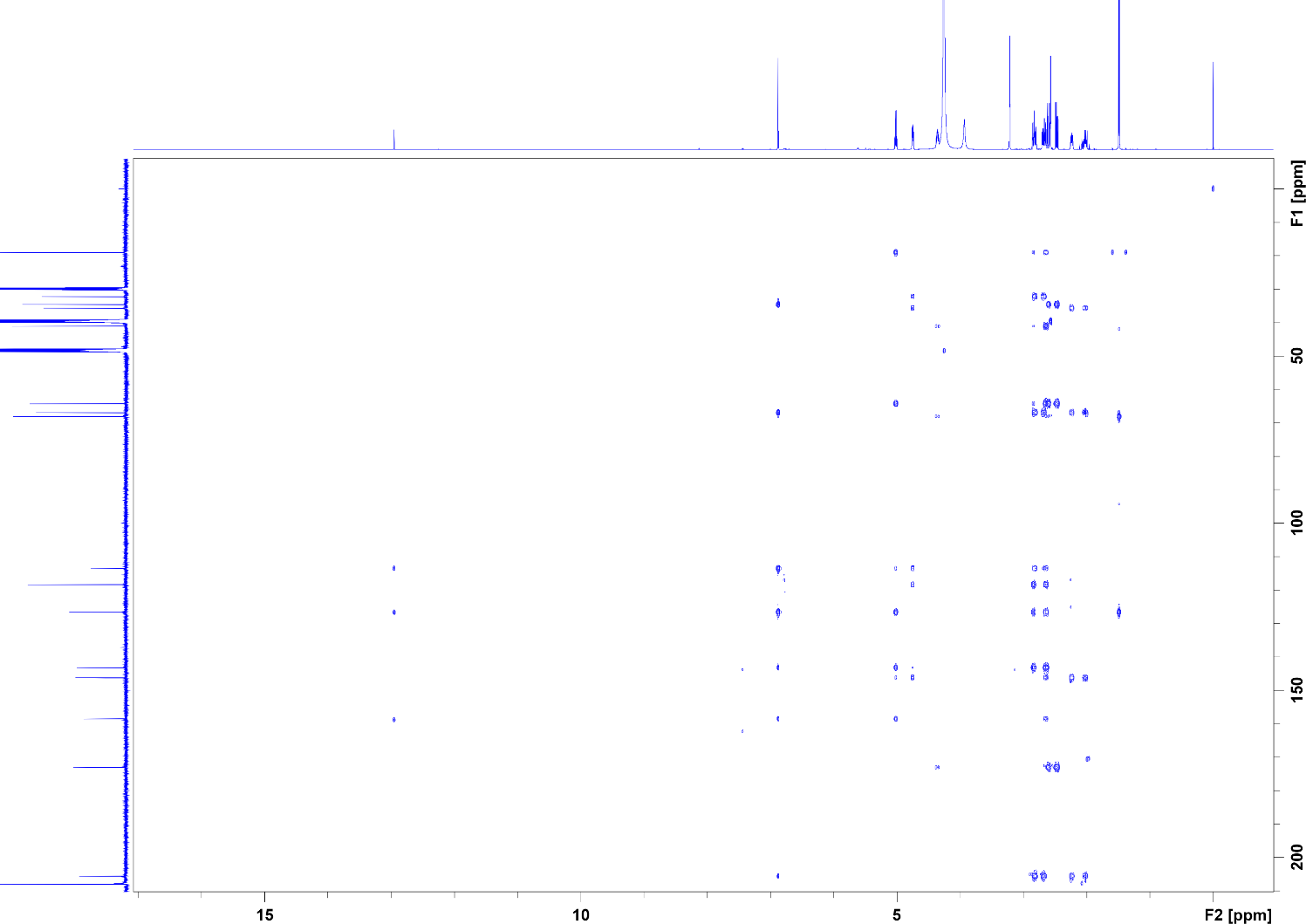
**

**Figure S14. HMBC spectrum of fogacin in CD_3_OD, DMSO-*d6* and acetone-*d6*.**

**
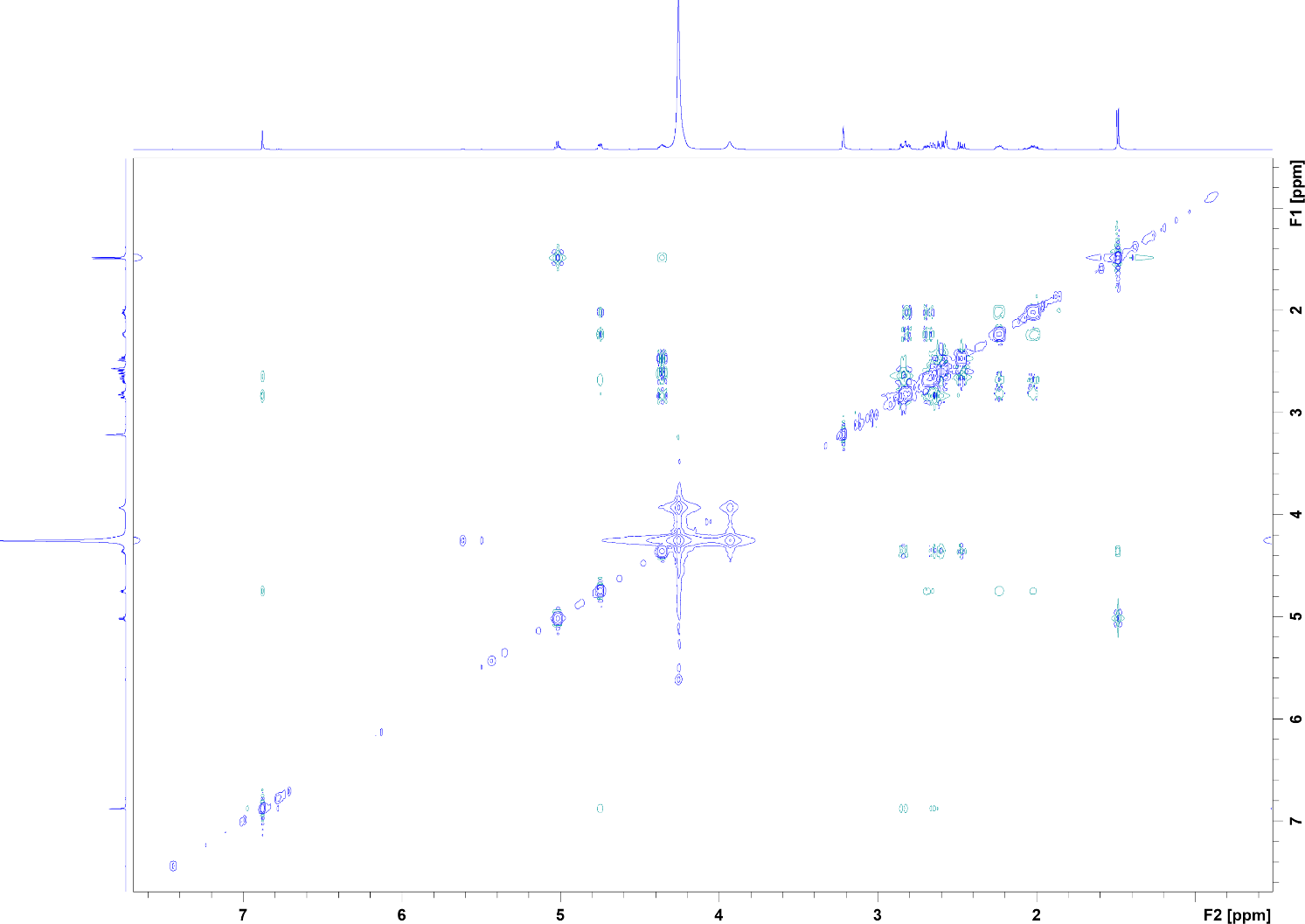
**

**Figure S15. NOESY spectrum of fogacin in CD_3_OD, DMSO-*d6* and acetone-*d6*.**


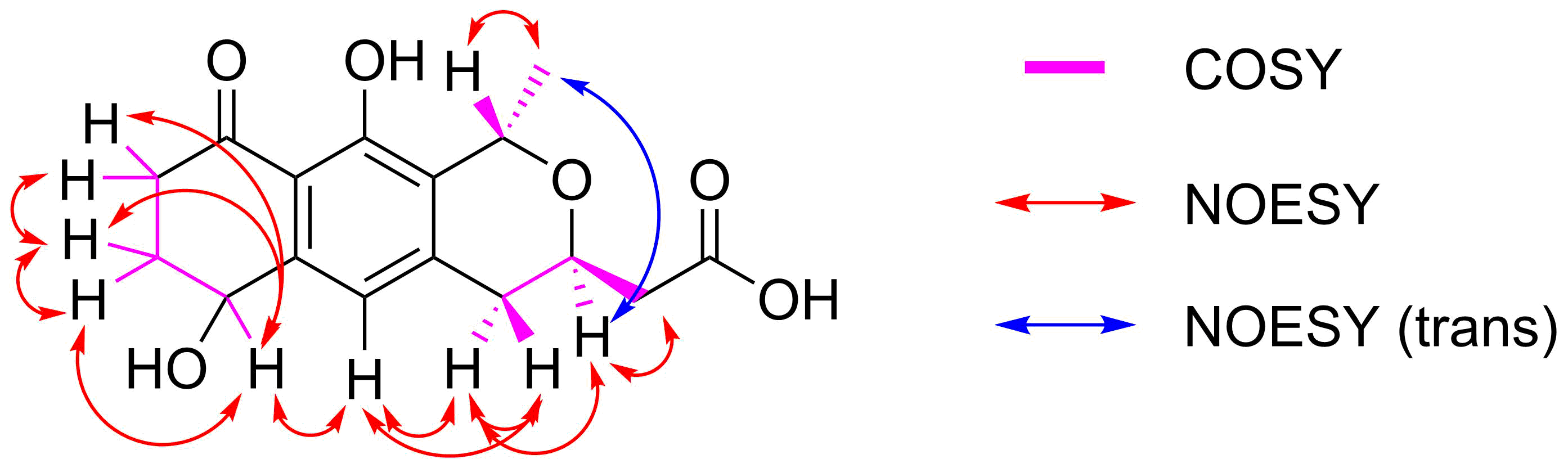


**Figure S16. COSY and NOESY correlations for fogacin.**

**
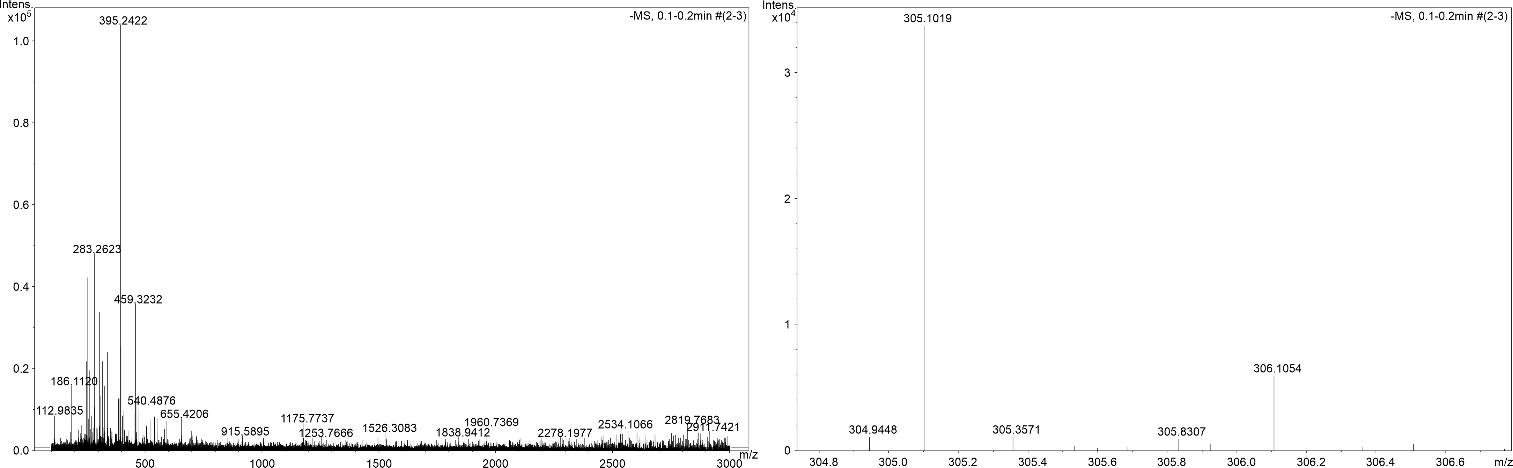
**

**Figure S17. HR-MS spectrum of fogacin.** ESI *m/z* [M-H]^-^, ESI^-^ obs. 305.1019, calc. 305.1131.


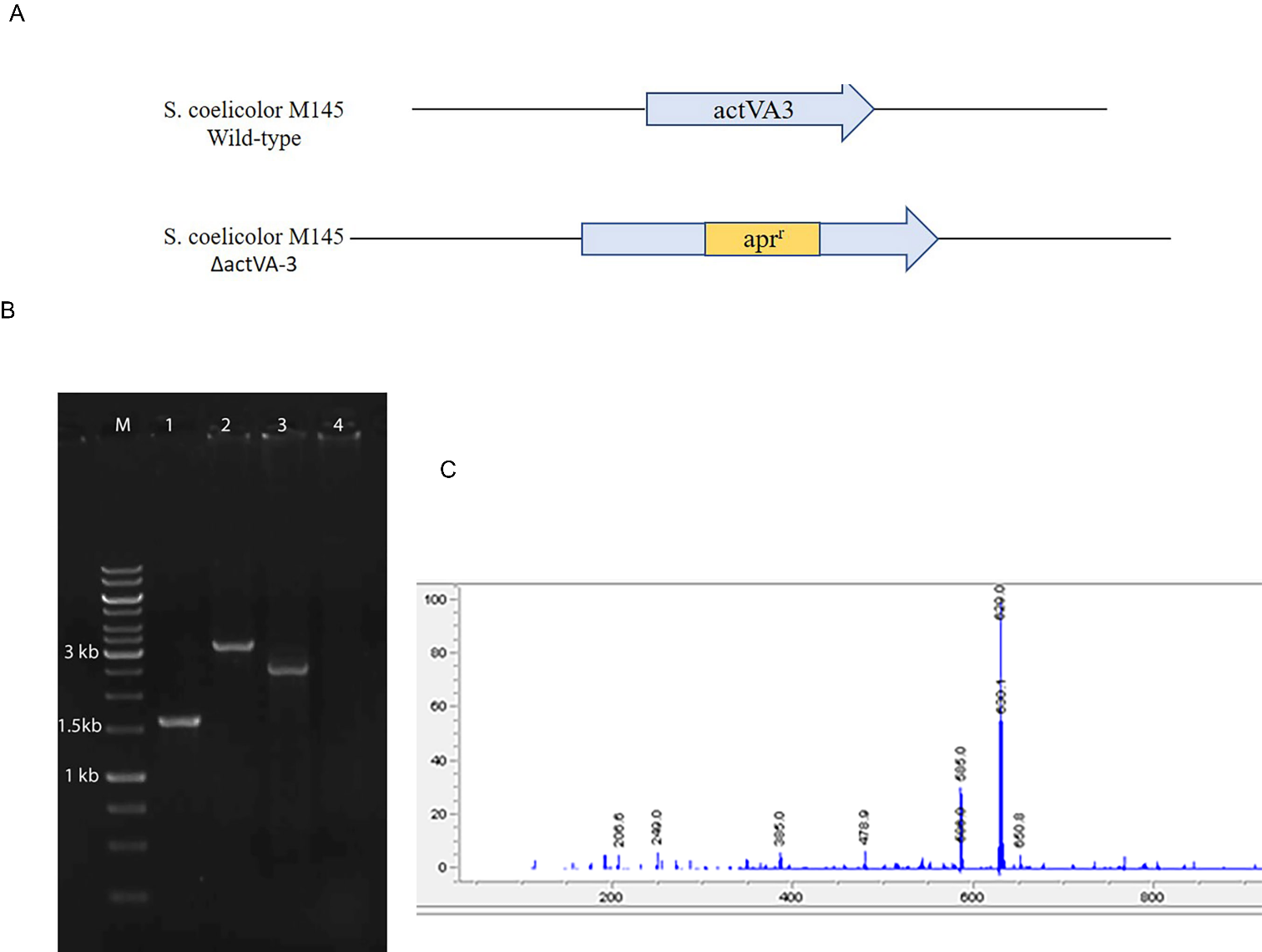


**Figure S18. Construction of the *S. coelicolor* mutant ∆actVA-3 by gene disruption with the apramycin resistance gene (apr^r^).** (A)The organizations of genes in the wild-type and ∆actVA-3, are shown. (B) Confirmation of the constructed *S. coelicolor* ∆actVA-3 mutant by PCR analysis. Lanes 1) and 2) The expected sizes of PCR products from the *S. coelicolor* mutant ∆actVA-3 using actVA3_ F and apr_R primers (1596 bp), and using actVA3_ F and actVA3_R (3176 bp). Lanes 3) and 4) The expected sizes of PCR products from the *S. coelicolor* wild type strain using actVA3_ F and actVA3_R (2176 bp), and using actVA3_ F and apr_R primers (no product expected). The DNA size marker in the M lane is a 1-kb DNA ladder. (C) LC-MS analysis the metabolite was analyzed as γ actinorhodin, with *m/z* value at 629.0 in a negative mode.

**Supplementary Tables**

**Table S1. Genomic potential of *S. arenae* DSM 40737 for production of secondary metabolites and their predicted novelty.** The naphthocyclinone biosynthetic gene cluster (BGC) is located within region 15.1 from 94,866 bp to 120,786 bp and is marked in red. Similarity is shown as the percentage of genes that have a significant BLAST hit between the predicted BGC and the most similar known cluster. Putatively novel BGCs are highlighted in bold. A distance score threshold above 900 was used as defined by the clustering of gene cluster families (GCF).

| **Region** | **Type** | **From** | **To** | **Length** | **Most Similar known cluster** | **Similarity** | **Completeness** | **Best hit** | **Distance** |
| --- | --- | --- | --- | --- | --- | --- | --- | --- | --- |
| **2.1** | **T3PKS** | **41,226** | **82,326** | **41,100** | -- | -- | **complete** | **GCF_01542** | **1105** |
| 4.1 | T3PKS, NRPS | 1 | 40,626 | 40,625 | teicoplanin | 20 | fragmented | GCF_01170 | 1590 |
| 4.2 | terpene | 51,239 | 76,804 | 25,565 | isorenieratene | 63 | complete | GCF_24312 | 120 |
| 5.1 | T2PKS | 1 | 33,191 | 33,190 | spore pigment | 83 | fragmented | GCF_13459 | 1417 |
| **6.1** | **NRPS** | **190,495** | **234,499** | **44,004** | **phosphonoglycans** | **3** | **complete** | **GCF_12505** | **1185** |
| 8.1 | RiPP-like | 72,351 | 83,625 | 11,274 | -- | -- | complete | GCF_00876 | 580 |
| 8.2 | terpene | 113,764 | 135,929 | 22,165 | geosmin | 100 | complete | GCF_02104 | 344 |
| 9.1 | terpene | 1 | 18,944 | 18,943 | hopene | 61 | fragmented | GCF_01018 | 859 |
| **11.1** | **terpene** | **120,231** | **141,175** | **20,944** | **borrelidin** | **5** | **complete** | **GCF_09908** | **1184** |
| 14.1 | T3PKS | 105,200 | 146,264 | 41,064 | herboxidiene | 8 | complete | GCF_00824 | 752 |
| **14.2** | **siderophore, betalactone** | **166,278** | **210,433** | **44,155** | **natamycin** | **9** | **complete** | **GCF_00811** | **1410** |
| 15.1 | T2PKS | 81,788 | 154,339 | 72,551 | granaticin | 35 | complete | GCF_24313 | 550 |
| 18.1 | NRPS | 1 | 4,747 | 4,746 | -- | -- | fragmented | GCF_00012 | 394 |
| 24.1 | terpene | 4,932 | 26,017 | 21,085 | albaflavenone | 100 | complete | GCF_18936 | 347 |
| **26.1** | **betalactone** | **24,634** | **64,675** | **40,041** | -- | -- | **complete** | **GCF_19779** | **1061** |
| **30.1** | **NRPS, T1PKS** | **89,698** | **139,727** | **50,029** | **tubulysin A-I** | **17** | **complete** | **GCF_11681** | **1561** |
| 32.1 | NRPS | 1 | 14,493 | 14,492 | -- | -- | fragmented | GCF_00012 | 372 |
| 37.1 | NRPS | 1 | 71,835 | 71,834 | scabichelin | 90 | fragmented | GCF_24311 | 726 |
| 29.1 | NRPS | 5,283 | 35,088 | 29,805 | feglymycin | 15 | fragmented | GCF_20494 | 1238 |
| **40.1** | **T2PKS** | **82,784** | **155,275** | **72,491** | **fluostatins M-Q** | **25** | **complete** | **GCF_14030** | **1011** |
| 41.1 | NRPS | 1 | 7,757 | 7,756 | deptomycin | 4 | fragmented | GCF_00012 | 497 |
| **43.1** | **melanin** | **50,519** | **61,028** | **10,509** | **melanin** | **60** | **complete** | **GCF_12505** | **1185** |
| 43.2 | siderophore | 179,585 | 191,354 | 11,769 | desferrioxamin B / E | 83 | complete | GCF_02111 | 689 |
| 47.1 | siderophore | 144,327 | 156,366 | 12,039 | -- | -- | complete | GCF_13384 | 636 |
| **52.1** | **phosphonate** | **78,156** | **119,016** | **40,860** | **dehydrofosmidomycin** | **15** | **complete** | **GCF_24309** | **927** |
| 63.1 | NAPAA | 121,155 | 155,021 | 33,866 | -- | -- | complete | GCF_00817 | 617 |
| 68.1 | T3PKS, butyrolactone | 86,396 | 127,580 | 41,184 | lactonamycin | 10 | complete | GCF_24315 | 0 |
| 72.1 | lanthipeptide-class-iii, RiPP-like | 5,204 | 32,875 | 27,671 | informatipeptin | 100 | complete | GCF_01977 | 553 |
| 72.2 | terpene | 68,405 | 89,436 | 21,031 | -- | -- | complete | GCF_09234 | 926 |
| 72.3 | NAPAA | 175,410 | 211,251 | 35,841 | stenothricin | 13 | complete | GCF_24310 | 641 |
| 73.1 | hglE-KS, T1PKS | 72,836 | 103,005 | 30,169 | -- | -- | fragmented | GCF_28220 | 1286 |
| 76.1 | lanthipeptide-class-iv | 7,660 | 30,596 | 22,936 | venezuelin | 100 | complete | GCF_01133 | 979 |
| 78.1 | NRPS | 1 | 33,915 | 33,914 | -- | -- | fragmented | GCF_00012 | 864 |
| 80.1 | NRPS | 1 | 17,324 | 17,323 | -- | -- | fragmented | GCF_00012 | 439 |
| 81.1 | NRPS | 1 | 19,856 | 19,855 | -- | -- | fragmented | GCF_00012 | 423 |
| 82.1 | melanin, terpene | 45,167 | 64,776 | 19,609 | melanin | 71 | complete | GCF_13966 | 785 |
| 82.2 | terpene | 131,130 | 150,989 | 19,859 | -- | -- | fragmented | GCF_12506 | 796 |
| **84.1** | **NRPS** | **19,772** | **62,906** | **43,134** | **oxalomycin B** | **9** | **complete** | **GCF_24162** | **1795** |
| 85.1 | siderophore, amglyccycl | 82,285 | 106,561 | 24,276 | cetoniacytone A | 12 | complete | GCF_19298 | 859 |
| 87.1 | ectoine | 81,091 | 91,489 | 10,398 | ectoine | 100 | complete | GCF_00822 | 808 |
| 91.1 | NRPS | 1 | 3,891 | 3,890 | -- | -- | fragmented | GCF_00012 | 394 |
| 92.1 | RiPP-like | 1,068 | 12,993 | 11,925 | -- | -- | complete | GCF_01142 | 862 |

**Table S2. NMR spectral data of α-naphthocyclinone acid recorded in CD_3_OD. ^1^H is recorded at 600 MHz and ^13^C at 151 MHz.** The signals are internally referenced to tetramethylsilane (TMS).


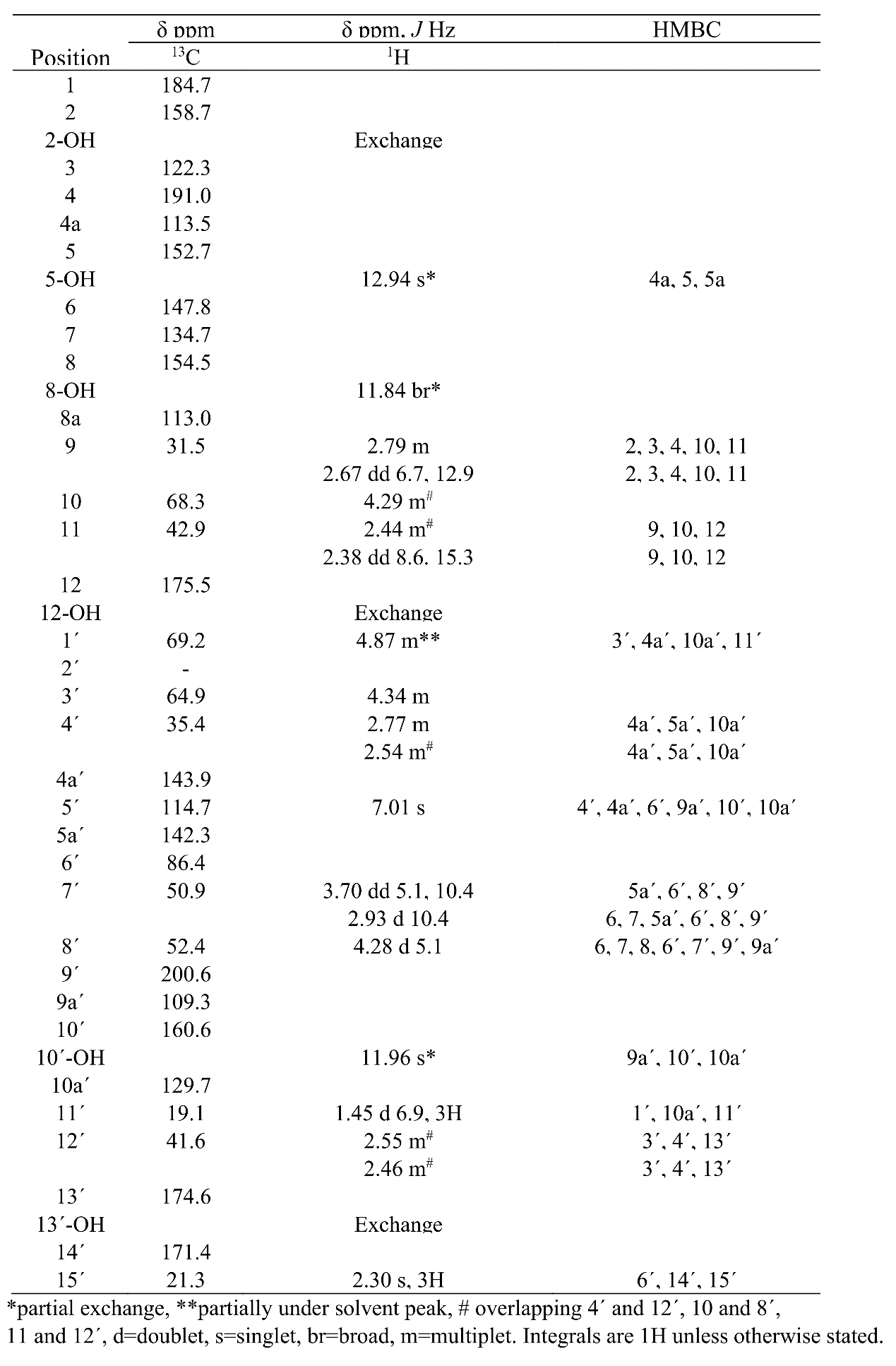


**Table S3. NMR spectral data of fogacin recorded in CD_3_OD, DMSO*-d6* and acetone*-d6*.**

**^1^H is recorded at 600 MHz and ^13^C at 151 MHz.** The signals are internally referenced to

TMS.


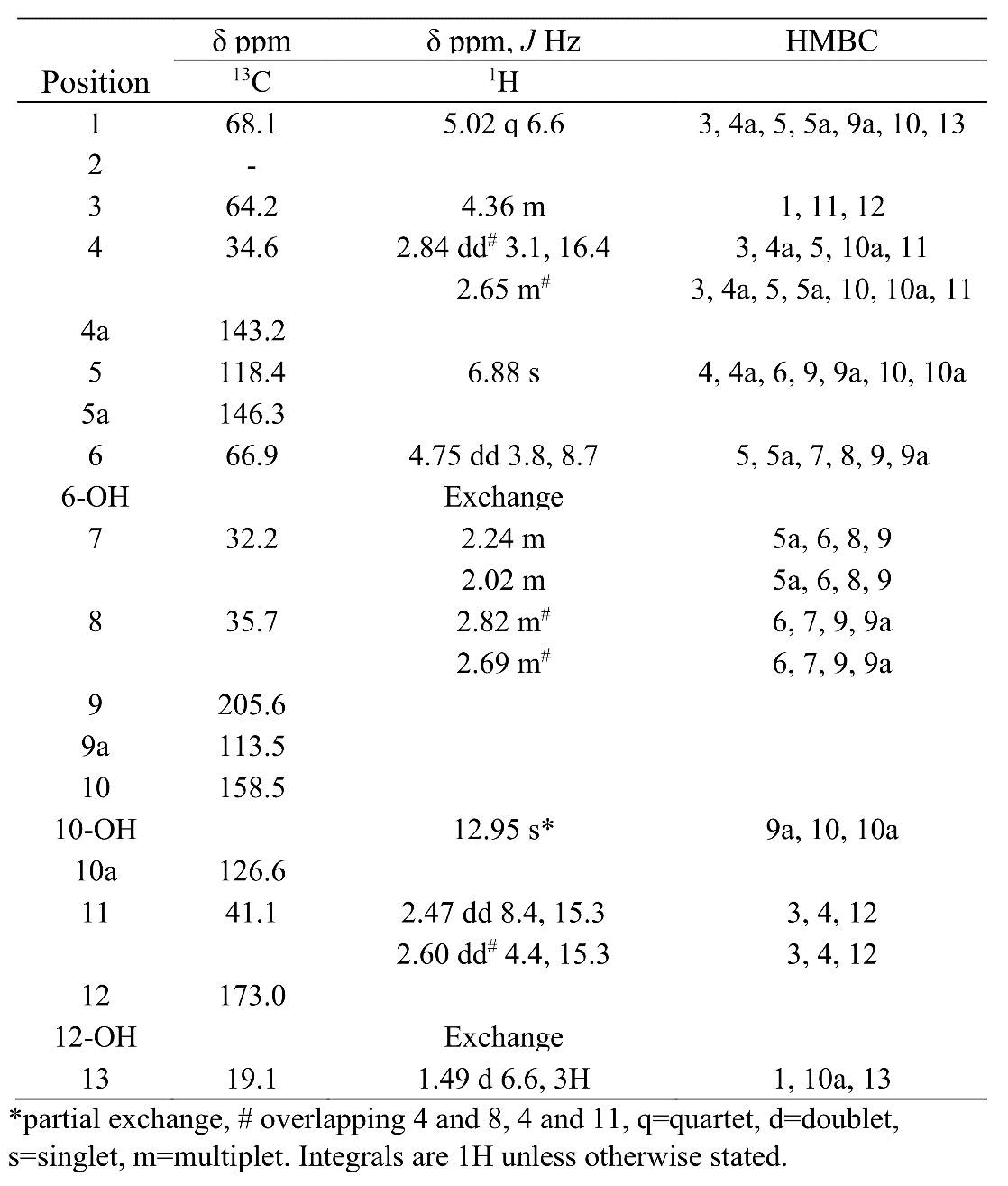


**References:**

Hopwood D A, Bidd J. B *et al*. *Genetic Manipulation of* Streptomyces*: A Laboratory Manual*, John Innes Foundation, **1985**.

Sambrock J, Fritsch EF, Maniatis T. *Molecular Cloning: A Laboratory Manual*, Cold Spring Harbor Laboratory Press, **1989**.

Kieser THDA, Bibb MJ *et al*. *Practical Streptomyces Genetics. 1 Edn. Norwich: The John Innes Foundation.*, **2000**.
